# Discrimination between Eggs from Stink Bugs Species in Europe Using MALDI-TOF MS

**DOI:** 10.3390/insects12070587

**Published:** 2021-06-28

**Authors:** Michael A. Reeve, Tim Haye

**Affiliations:** 1 CABI, Bakeham Lane, Egham, Surrey TW20 9TY, UK; 2 CABI, Rue des Grillons 1, CH-2800 Delémont, Switzerland; t.haye@cabi.org

**Keywords:** stink bugs, MALDI-TOF MS, *Halyomorpha halys*

## Abstract

**Simple Summary:**

Recent globalization of trade and travel has led to the introduction of exotic insects into Europe, including the brown marmorated stink bug (*Halyomorpha halys*)—a highly polyphagous pest with more than 200 host plants, including many agricultural crops. Unfortunately, farmers and crop-protection advisers finding egg masses of stink bugs during crop scouting frequently struggle to identify correctly the species based on their egg masses, and easily confuse eggs of the invasive *H. halys* with those of other (native) species. To this end, we have investigated using matrix-assisted laser desorption and ionization time-of-flight mass spectrometry (MALDI-TOF MS) for rapid, fieldwork-compatible, and low-reagent-cost discrimination between the eggs of native and exotic stink bugs.

**Abstract:**

In the current paper, we used a method based on stink bug egg-protein immobilization on filter paper by drying, followed by post-(storage and shipping) extraction in acidified acetonitrile containing matrix, to discriminate between nine different species using MALDI-TOF MS. We obtained 87 correct species-identifications in 87 blind tests using this method. With further processing of the unblinded data, the highest average Bruker score for each tested species was that of the cognate reference species, and the observed differences in average Bruker scores were generally large and the errors small except for *Capocoris fuscispinus*, *Dolycoris baccarum*, and *Graphosoma italicum*, where the average scores were lower and the errors higher relative to the remaining comparisons. While we observed clear discrimination between the nine species using this method, *Halyomorpha halys* and *Piezodorus lituratus* were more spectrally related than the other pairwise comparisons.

## 1. Introduction

Within the suborder Heteroptera (true bugs), there are more than 36,000 described species worldwide and among those there are over 4000 species belonging to the family Pentatomidae (stink bugs). The large majority of stink bugs are herbivores and, because of their highly polyphagous nature and ability to survive unfavorable conditions, many species are considered to be economically important pests [1]. Increased human trade and transport over the last few decades have resulted in the growing spread of invasive species [2], including the pentatomid pests *Halyomorpha halys* (Stål), *Nezara viridula* (L.), *Bagrada hilaris* (Burmeister), *Murgantia histrionica* (Hahn), and *Piezodorus guildiniii* (Westwood) [3]. In Europe and the Caucasus in particular, the widespread *H. halys* has generated a lot of attention due to severe crop losses caused by the bug in Northern Italy and Georgia [4,5]. While keys for identifying adult stink bugs based on morphological characteristics are available [6,7,8], there is still a lack of such keys for the eggs of stink bugs in the above regions. Given this, an alternative method for species identification could be matrix-assisted laser desorption and ionization time-of-flight mass spectrometry (MALDI-TOF MS), a rapid method for the analysis of protein-containing samples in which mass-over-charge ratios are derived from the time-of-flight of singly charged proteins [9,10] accelerated by means of an electrical field into a tube held at high vacuum [11,12]. MALDI-TOF MS is often used with the acid-soluble and highly expressed subset of the proteome, which including many ribosomal proteins [12].

Numerous MALDI-TOF MS sample preparation methods have been developed for use with microorganisms [11,13,14,15,16], along with insects [16] and plants [16,17,18]. Regardless of the method used for sample preparation; however, MALDI-TOF MS requires relatively fresh biological material—the analyzed proteins must not have undergone significant degradation. To overcome this limitation, Reeve and Buddie, have developed a simple and inexpensive filter-paper-based method for the practical storage of field-sample proteins [19]. This was originally developed for use with plant material but has since been extended in scope to cover seeds [20,21] and insects [22]. In each of these method variants, the underlying rationale is to immobilize the proteins from lysed cells onto filter paper by drying thoroughly such that the proteins of interest and any proteases will remain spatially separated while dry. After drying, the filter papers may be stored and shipped dry, without any requirement for low temperatures [19], followed by acid-extraction of proteins from the paper and MALDI-TOF MS analysis

In the current paper, we have used the method variant of Reeve and Seehausen [22] with the eggs of stink bugs to discriminate between nine different species present in Europe, including the invasive *H. halys* and *N. viridula*, using MALDI-TOF MS.

## 2. Materials and Methods

The *H. halys* colony was originally established in 2017 from individuals collected in Basel, Switzerland. Adults of other stink bugs species were collected in early spring by visual inspection or plant beating from their herbaceous and arboreous host plants in the Jura mountains, NW Switzerland. Species were identified using the keys by [6,7,8]. The insects were maintained at 23 °C, 70% RH, and a 16L:8D photoperiod and provided with potted broad bean plants, bramble branches, apples, hazelnuts, and green beans, which were replaced once per week. Newly laid egg masses were collected from the screen of the gauze cages on a daily basis. The stink bug species and samples used in this this study are shown in Table 1.

Ethanol (≥99.8%), α-cyano-4-hydroxycinnamic acid (HCCA) matrix (TLC grade, ≥98%), acetonitrile (LC-MS-grade), trifluoroacetic acid (TFA) (ReagentPlus^®^-grade, 99%), and Whatman^®^ qualitative filter paper (Grade 1, 90 mm circles), were purchased from Sigma (Gillingham, UK). Water (CHROMASOLV^TM^ LC-MS-grade) was purchased from Fluka (Loughborough, UK).

For each stink bug species (*n* = 9), 10 egg masses were randomly selected for testing. Eggs used for testing were not older than 24 h. For *P. prasina* (PP) only seven egg masses were available. From each of the 87 egg masses, two eggs were taken off, crushed onto filter paper (approximately 10 mm × 4 mm), dried, and stored as described in [22]. One of the eggs was labeled with a species code (e.g., CF1A) and the second one was labeled with a code (e.g., X1) that did not indicate the species (“blind sample”).

Acid-soluble proteins were extracted by immersing the paper in 100 µL of (11 mg/mL HCCA matrix in 65% (*v/v*) acetonitrile, 2.5% (*v/v*) TFA, and 32.5% (*v/v*) water), capping the tube, briefly vortexing, soaking for 30 min, and vortexing again. One microliter of the supernatant was then pipetted onto the Bruker sample plate, air dried, and loaded into the spectrometer.

Mass spectrometry covering the range 2 kDa to 20 kDa was carried out using a Bruker Microflex LT linear-mode instrument running the MALDI Biotyper 4.0 applications (Bruker Daltonik, Bremen, Germany), with settings, plates, and calibration as described in [22]. Database entries were made as single-spectra MSPs (main spectra) using the Bruker Online Client software suite (Version 4.0.19, Bruker Daltonik, Bremen, Germany), using the manufacturer’s standard settings. For spectral comparisons, Bruker identification scores were derived using the standard Bruker algorithm described in [22]. Bruker scores between 2.3 and 3.0 indicate very close relatedness, scores between 2.0 and 2.3 indicate close relatedness, scores between 1.7 and 2.0 indicate intermediate relatedness, and scores below 1.7 indicate low relatedness.

Ten “reference” sample preparations (seven for PP) were carried out as indicated for each of the nine species, from which a database of 87 reference spectra was generated. For blind testing, randomized and numbered tubes were supplied containing ten samples of each species (seven for PP), giving a panel of 87 “test” samples. For spectral comparison, all 87 blind-test samples were compared against the database of all 87 reference spectra and Bruker identification scores were generated for all 7569 comparisons.

For the blind-test samples, the highest Bruker score from the 87 reference samples was used to make the identification call. After confirming the accuracy of the 87 blind-test identifications by unblinding the data, the spectral comparison data were sorted alphabetically by reference-sample name for each test sample (now identified). These were then ordered alphabetically by test-sample identity to generate an 87 × 87 matrix of test samples against reference samples from which average values and standard errors for all 81 pairwise species comparisons were calculated.

## 3. Results

The MALDI-TOF MS spectra for the 87 reference samples are shown in Figures S1–S9. For ease of comparison, the replicate-1 egg-protein spectra for the nine species of stink bugs are shown in Figure 1.

**Figure 2:**
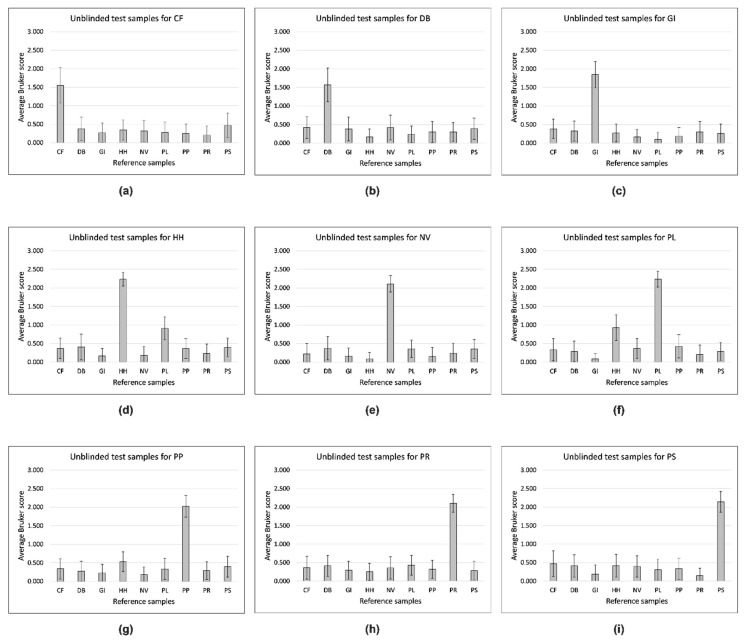
Average Bruker score values and standard errors for all 81 pairwise species comparisons. CF = *Carpocoris fuscispinus*, DB = *Dolycoris baccarum*, GI = *Graphosoma italicum*, HH = *Halyomorpha halys*, NV = *Nezara viridula*, PL = *Piezodorus lituratus*, PP = *Palomena prasina*, PR = *Pentatoma rufipes*, and PS = *Peribalus strictus*.

Figure 2 shows visibly distinct replicate-1 MALDI-TOF MS egg-protein spectra from the nine stink bug species. For blind testing, in which 87 test-sample spectra were compared against a database of all 87 reference spectra, the highest Bruker scores from the 87 reference samples was used to make the identification. After confirming the accuracy of all 87 blind-test identifications by unblinding the data, the spectral comparison data (sorted alphabetically by reference-sample name for each test sample and ordered alphabetically by test-sample identity) are shown in Tables S1–S3. From the data in Tables S1–S3, average Bruker score values and standard errors for all 81 pairwise species comparisons were calculated, and are shown in Figure 2 and Tables S4 and S5.

Figure 2 shows that the highest average Bruker score for each tested species is that of the cognate reference species. The differences between average Bruker scores are generally large and the errors small except for *C. fuscispinus* (CF), *D. baccarum* (DB), and *G. italicum* (GI) (Figure 2a–c), where the average scores are lower and the errors higher relative to the remaining comparisons (Figure 2d–i). While there is clear discrimination between the nine species using this method, *H. halys* (HH) and *P. lituratus* (PL) are more spectrally related than the other pairwise comparisons.

## 4. Discussion

**Figure 3:**
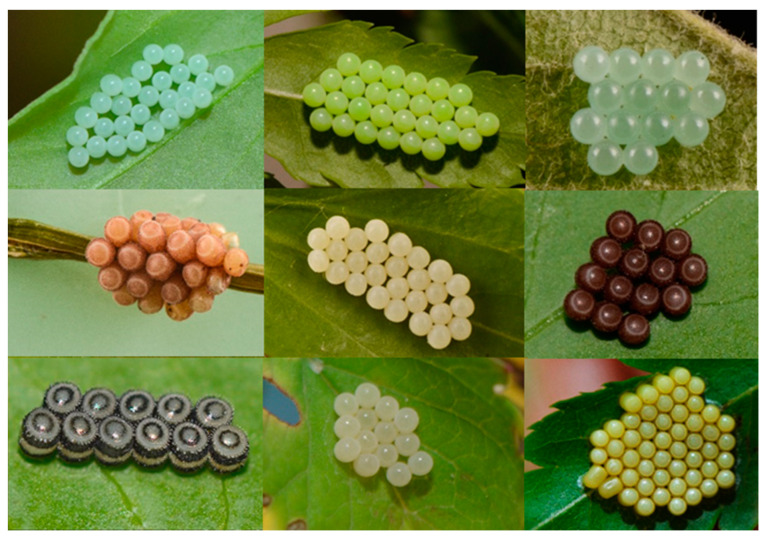
Egg masses of common stink bugs that are easily confused (always from left to right): **top row**: *Halyomorpha halys*, *Palomena prasina*, *Pentatoma rufipes*; **middle row**: *Dolycoris baccarum*, *Graphosoma italicum*, *Carpocoris fuscispinus*; **bottom row**: *Piezodorus lituratus*, *Peribalus strictus, Nezara viridula*.

In pest management, the correct identification of pest species is a key element. However, farmers and crop-protection advisers, finding egg masses of stink bugs during crop scouting, often struggle to identify correctly the species based on their egg masses and easily confuse eggs of the invasive *H. halys* with those of other native pentatomid pests such as *P. prasina* and *P. rufipes* (Figure 3). To date, identification to species level relies on molecular barcoding methods or the time-consuming rearing of eggs to the adult stage.

MALDI-TOF MS is a rapid and simple method, with generally low reagent costs ([16], Discussion section), or the analysis of protein-containing samples but which requires relatively fresh biological material because the analyzed proteins must not have undergone significant degradation—one of the few limitations of the method. Reeve and Buddie originally reported a convenient and cheap filter-paper-based method for the storage without degradation of plant proteins for later MALDI-TOF MS analysis [19], which has subsequently been adapted for use with seeds [20,21] and insects [22] and in the current paper, we have successfully adapted this methodology for use with the eggs of stink bugs (with the ambient-temperature shipping of the samples immobilized on filter paper from Switzerland to the UK after preparation and storage). Once again, as described in detail the Discussion section of [22], we have used an approach for high-resolution spectral comparisons that departs slightly from the standard Bruker ‘MSP’ method, and we have again used multiple sample replicates (rather than pipetting and/or laser-shot replicates) to accommodate better the real-life variances in the samples and analytical steps.

For simple testing according to the highest-scoring spectral comparison in the reference database constructed for this study, we obtained 87 correct identifications in 87 blind tests, each with nine possible outcomes—a probability of 1 in 10^83^ of occurring by chance, and a clear validation for the use of MALDI-TOF MS analysis of egg proteins to discriminate between stink bug species using this methodology. The above (while of great value and simplicity) does not however fully disclose sample-to-sample spectral variance, which is apparent from the replicate spectra shown in Figures S1–S9, particularly in Figures S1–S3 for *C. fuscispinus*, *D. baccarum,* and *G. italicum* respectively, where this variance results in the lower average Bruker scores and higher errors seen in Figure 2a–c. The cause of this higher variance may be biological or technical, and will be investigated in future studies. Finally, while this is a study aimed primarily at discriminating between stink bug species through their egg proteins, any observed spectral similarity is also of interest, which is noticeable between *H. halys* and *P. lituratus*, which belong to the same subfamily (Pentatominae), but two different tribes, Cappaeini and Piezodorini, respectively.

## 5. Conclusions

We successfully employed a method based on stink bug egg-protein immobilizations on filter paper by drying, followed by post-(storage and shipping) extraction in acidified acetonitrile containing matrix, to discriminate between nine different species using MALDI-TOF MS. With further processing of the unblinded data, the highest average Bruker score for each tested species was that of the cognate reference species, and the observed differences on average Bruker scores were generally large and the errors small except for *C. fuscispinus*, *D. baccarum*, and *G. italicum*, where the average scores were lower and the errors higher relative to the remaining comparisons. While we have observed clear discrimination between the nine species using this method, *H. halys* and *P. lituratus* were observed to be more spectrally related than the other pairwise comparisons. After the proof of principle that egg masses of different stink bugs species can be distinguished with MALDI-TOF MS, in a next step we will include egg masses parasitized by different scelionid and eupelmid wasps, which frequently attack stink bug egg masses in the field [23] and may influence the outcome of the analysis significantly. In addition, it is planned also to test if species belonging to the same genus can be easily separated and if the protein profile is changing with the development of the embryos by analyzing egg masses of different ages.

## Figures and Tables

**Figure 1:**
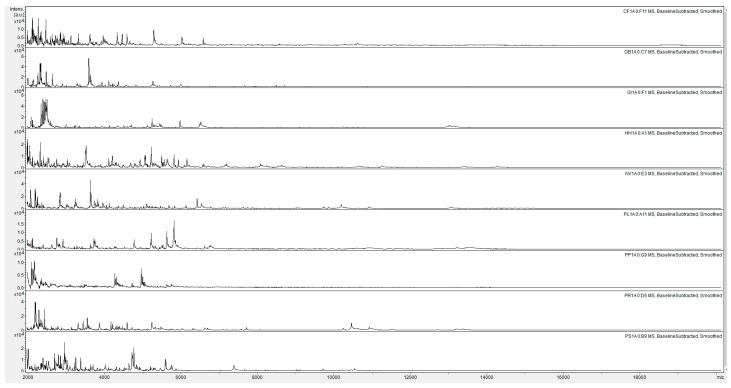
Replicate-1 MALDI-TOF MS spectra of acid-soluble egg proteins from nine species of stink bugs with, from top to bottom, CF = *Carpocoris fuscispinus*, DB = *Dolycoris baccarum*, GI = *Graphosoma italicum*, HH = *Halyomorpha halys*, NV = *Nezara viridula*, PL = *Piezodorus lituratus,* PP = *Palomena prasina*, PR = *Pentatoma rufipes*, and PS = *Peribalus strictus*. Spectra are shown baseline-subtracted, smoothed, with y-axis autoscaling, and covering the mass range 2 kDa to 20 kDa (with x-axis scale increments of 2 kDa).

**Table 1:** Stink bug species and samples used in the current study.

Species	Status	Number of Samples	Pest On
*Carpocoris fuscispinus* (Boheman)	native	10	-
*Dolycoris baccarum* (L.)	native	10	berry crops
*Graphosoma lineatum* (L.)	native	10	-
*Halyomorpha halys* (Stål)	exotic	10	fruit crops, vegetables, nuts
*Nezara viridula* (L.)	exotic	10	vegetables, fruit crops
*Palomena prasina* (L.)	native	7	nuts, fruit crops
*Pentatoma rufipes* (L.)	native	10	fruit crops
*Peribalus strictus* (F.)	native	10	-
*Piezodorus lituratus* (F.)	native	10	-

## Data Availability

Original spectral data held on the Bruker Microflex PC is available on request.

## Supplementary Materials

The following are available online at https://www.mdpi.com/article/10.3390/insects12070587/s1; Figures S1: MALDI-TOF MS spectra of acid-soluble egg proteins from CF = Capocoris fuscispinus with, from top to bottom, replicates 1 to 10. Spectra are shown baseline-subtracted, smoothed, with y-axis autoscaling, and covering the mass range 2 kDa to 20 kDa (with x-axis scale increments of 2 kDa); Figure S2: MALDI-TOF MS spectra of acid-soluble egg proteins from DB = Dolycoris baccarum with, from top to bottom, replicates 1 to 10. Spectra are shown baseline-subtracted, smoothed, with y-axis autoscaling, and covering the mass range 2 kDa to 20 kDa (with x-axis scale increments of 2 kDa); Figure S3: MALDI-TOF MS spectra of acid-soluble egg proteins from GI = Graphosoma italicum with, from top to bottom, replicates 1 to 10. Spectra are shown baseline-subtracted, smoothed, with y-axis autoscaling, and covering the mass range 2 kDa to 20 kDa (with x-axis scale increments of 2 kDa); Figure S4: MALDI-TOF MS spectra of acid-soluble egg proteins from HH = Halyomorpha halys with, from top to bottom, replicates 1 to 10. Spectra are shown baseline-subtracted, smoothed, with y-axis autoscaling, and covering the mass range 2 kDa to 20 kDa (with x-axis scale increments of 2 kDa); Figure S5: MALDI-TOF MS spectra of acid-soluble egg proteins from NV = Nezara viridula with, from top to bottom, replicates 1 to 10. Spectra are shown baseline-subtracted, smoothed, with y-axis autoscaling, and covering the mass range 2 kDa to 20 kDa (with x-axis scale increments of 2 kDa); Figure S6: MALDI-TOF MS spectra of acid-soluble egg proteins from PL = Piezedorus lituratus with, from top to bottom, replicates 1 to 10. Spectra are shown baseline-subtracted, smoothed, with y-axis autoscaling, and covering the mass range 2 kDa to 20 kDa (with x-axis scale increments of 2 kDa); Figure S7: MALDI-TOF MS spectra of acid-soluble egg proteins from PP = Palomena prasina with, from top to bottom, replicates 1 to 7. Spectra are shown baseline-subtracted, smoothed, with y-axis autoscaling, and covering the mass range 2 kDa to 20 kDa (with x-axis scale increments of 2 kDa); Figure S8: MALDI-TOF MS spectra of acid-soluble egg proteins from PR = Pentatoma rufipes with, from top to bottom, replicates 1 to 10. Spectra are shown baseline-subtracted, smoothed, with y-axis autoscaling, and covering the mass range 2 kDa to 20 kDa (with x-axis scale increments of 2 kDa); Figure S9: MALDI-TOF MS spectra of acid-soluble egg proteins from PS = Peribalus strictus with, from top to bottom, replicates 1 to 10. Spectra are shown baseline-subtracted, smoothed, with y-axis autoscaling, and covering the mass range 2 kDa to 20 kDa (with x-axis scale increments of 2 kDa); Table S1: Bruker scores for comparisons between reference spectra (rows) and blind-test spectra (columns) subsequently identified as Capocoris fuscispinus, Dolycoris baccarum, and Graphosoma italicum; Table S2: Bruker scores for comparisons between reference spectra (rows) and blind-test spectra (columns) subsequently identified as Halyomorpha halys, Nezara viridula, and Piezedorus lituratus; Table S3: Bruker scores for comparisons between reference spectra (rows) and blind-test spectra (columns) subsequently identified as Palomena prasina, Pentatoma rufipes, and Peribalus strictus; Table S4: Average Bruker scores for all 81 pairwise species comparisons; Table S5: Standard errors of Bruker scores for all 81 pairwise species comparisons.
