# Supplementary material for: Discrimination between Eggs from Stink Bugs Species in Europe Using MALDI-TOF MS
Source: Insects. 2021 Jun 28;12(7):587. doi: 10.3390/insects12070587 (PMC8303754; doi:10.3390/insects12070587)
Supplement: Supplementary file 1 [file insects-12-00587-s001.zip › insects-1255073-supplementarym.pdf]

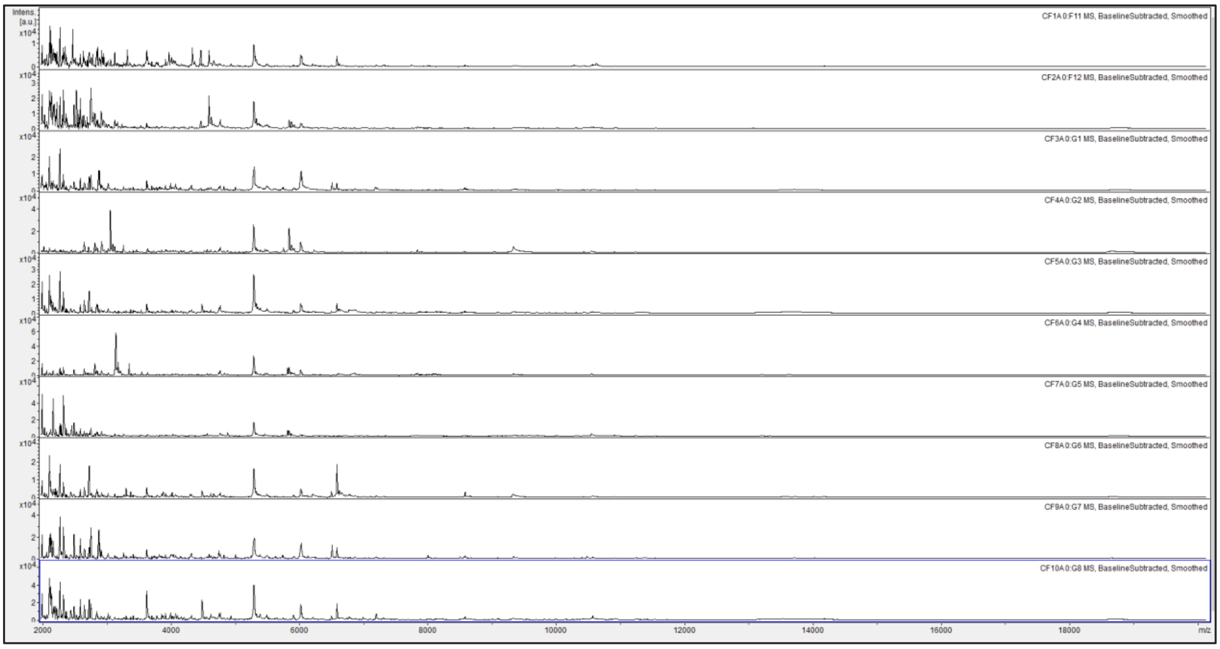

Figure S1. MALDI-TOF MS spectra of acid-soluble egg proteins from CF = *Capocoris fuscispinus* with, from top to bottom, replicates 1 to 10. Spectra are shown baseline-subtracted, smoothed, with y-axis autoscaling, and covering the mass range 2 kDa to 20 kDa (with x-axis scale increments of 2 kDa).

Figure S2

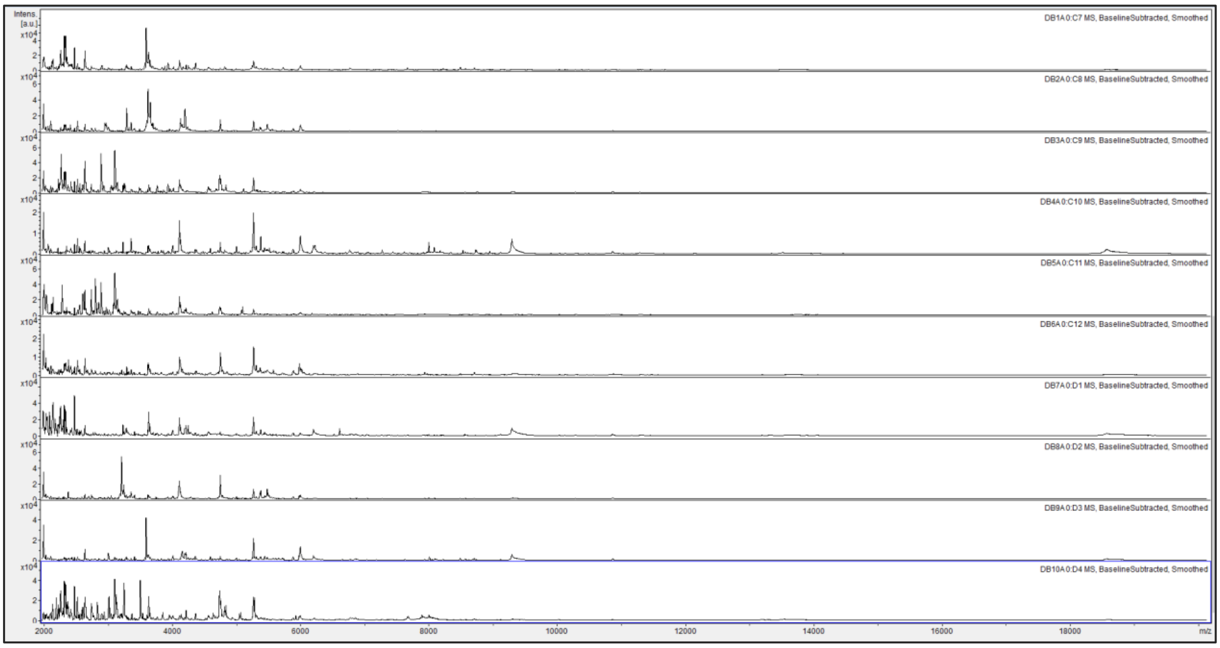

Figure S2 MALDI-TOF MS spectra of acid-soluble egg proteins from DB = *Dolycoris baccarum* with, from top to bottom, replicates 1 to 10. Spectra are shown baseline-subtracted, smoothed, with y-axis autoscaling, and covering the mass range 2 kDa to 20 kDa (with x-axis scale increments of 2 kDa).

Figure S3

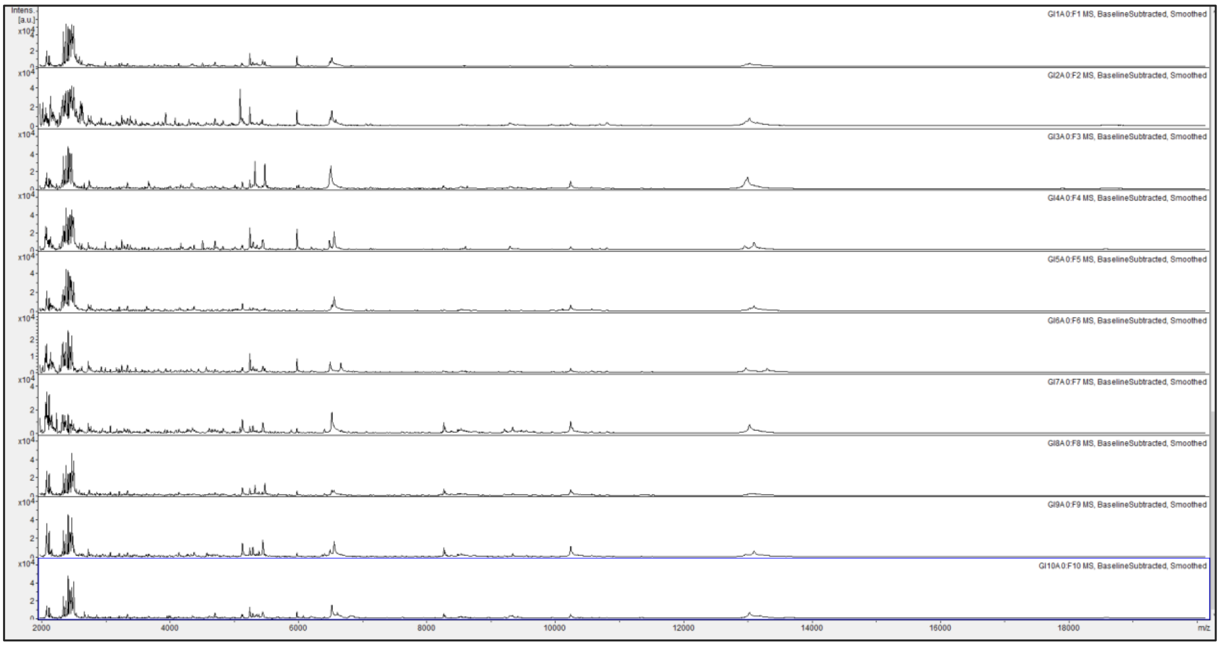

Figure S3 MALDI-TOF MS spectra of acid-soluble egg proteins from GI = *Graphosoma italicum* with, from top to bottom, replicates 1 to 10. Spectra are shown baseline-subtracted, smoothed, with y-axis autoscaling, and covering the mass range 2 kDa to 20 kDa (with x-axis scale increments of 2 kDa).

Figure S4

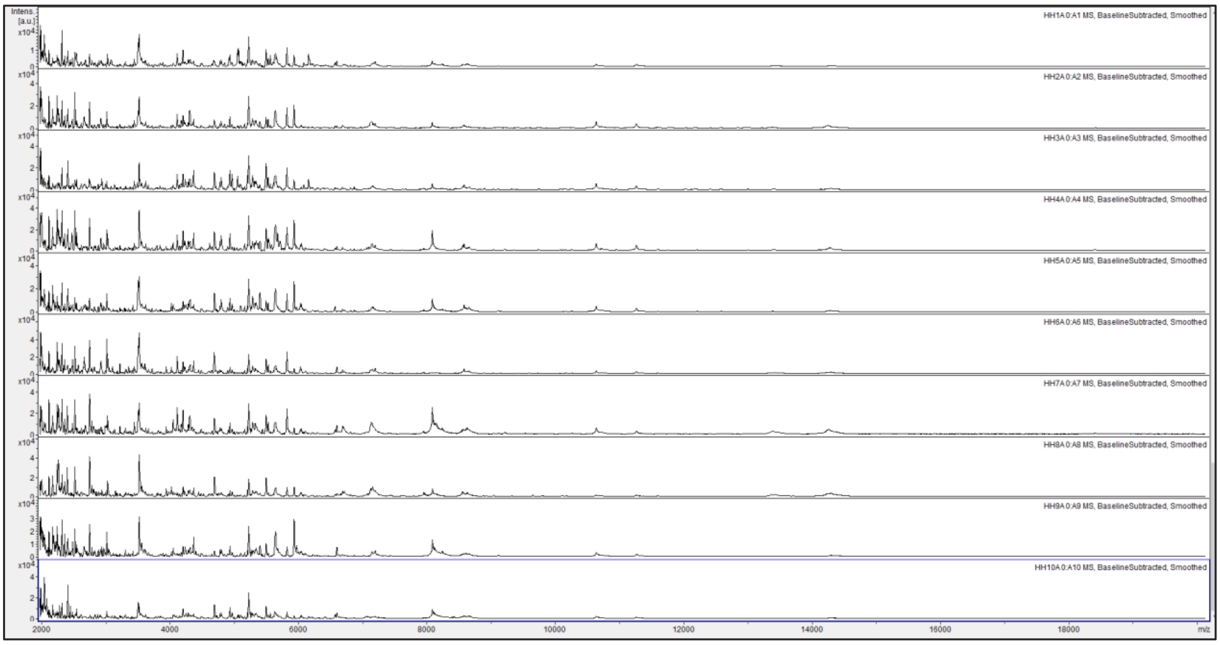

Figure S4 MALDI-TOF MS spectra of acid-soluble egg proteins from HH = *Halyomorpha halys* with, from top to bottom, replicates 1 to 10. Spectra are shown baseline-subtracted, smoothed, with y-axis autoscaling, and covering the mass range 2 kDa to 20 kDa (with x-axis scale increments of 2 kDa).

Figure S5

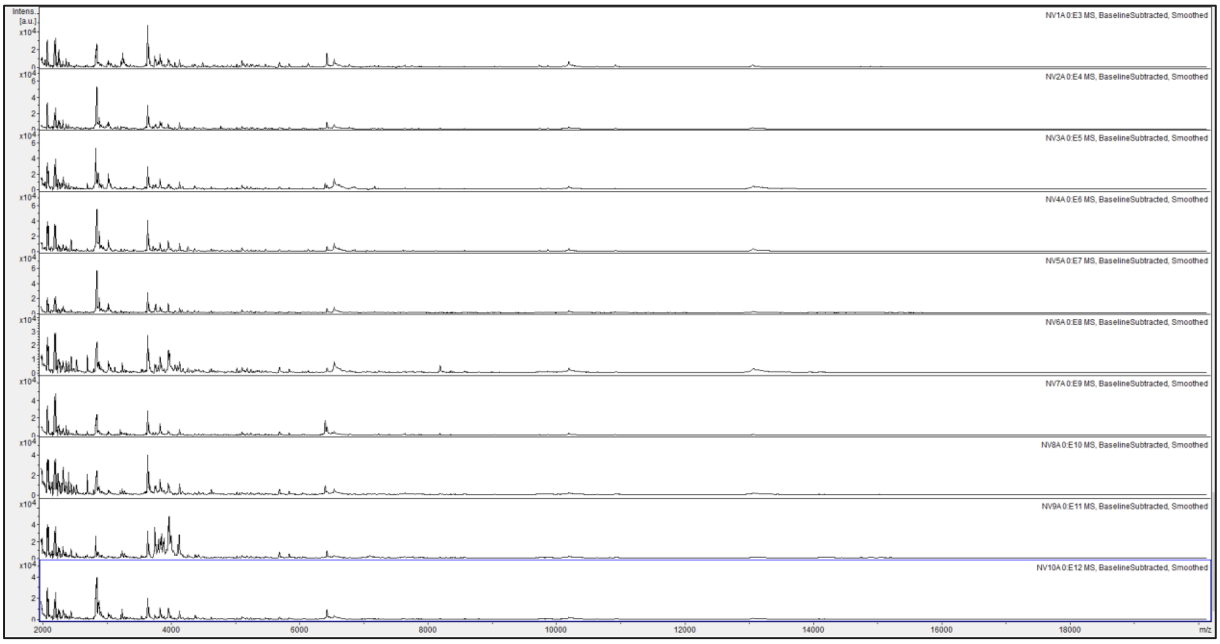

Figure S5 MALDI-TOF MS spectra of acid-soluble egg proteins from NV = *Nezara viridula* with, from top to bottom, replicates 1 to 10. Spectra are shown baseline-subtracted, smoothed, with y-axis autoscaling, and covering the mass range 2 kDa to 20 kDa (with x-axis scale increments of 2 kDa).

Figure S6

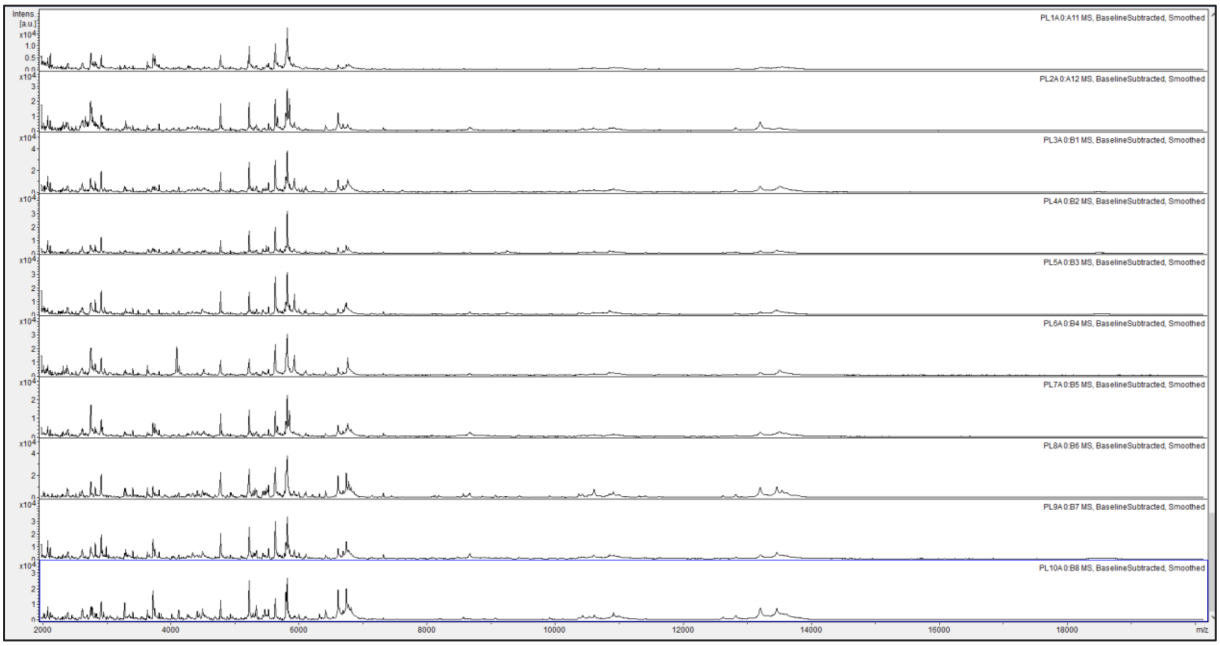

Figure S6 MALDI-TOF MS spectra of acid-soluble egg proteins from PL = *Piezedorus lituratus* with, from top to bottom, replicates 1 to 10. Spectra are shown baseline-subtracted, smoothed, with y-axis autoscaling, and covering the mass range 2 kDa to 20 kDa (with x-axis scale increments of 2 kDa).

Figure S7

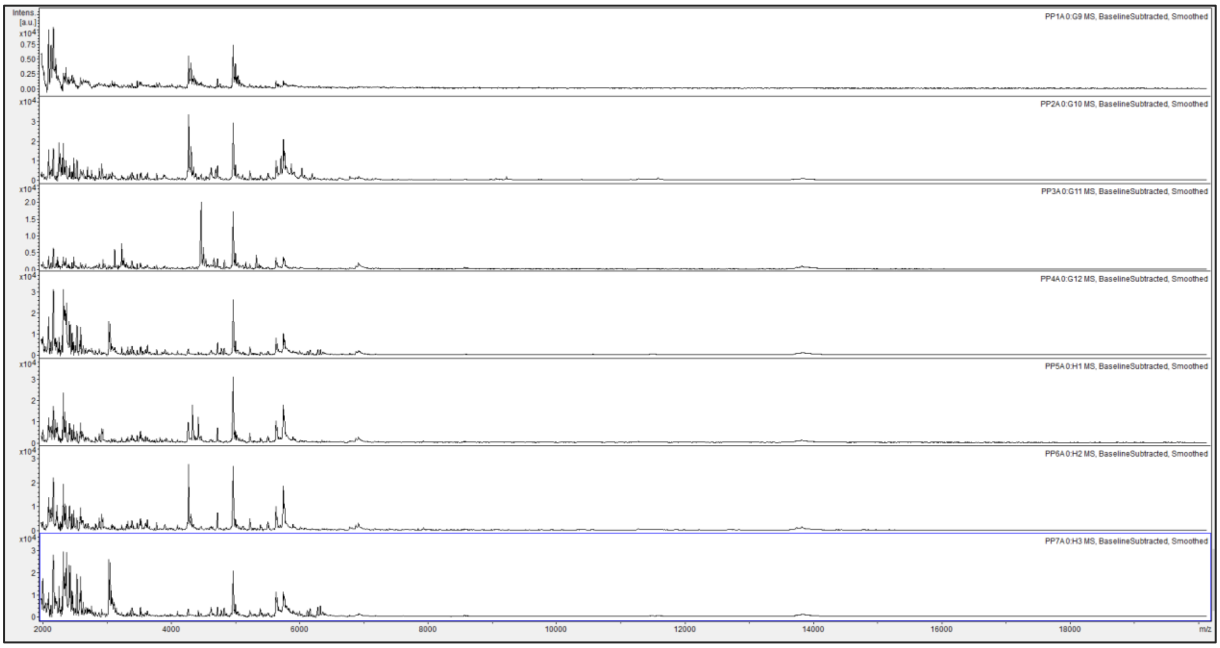

Figure S7 MALDI-TOF MS spectra of acid-soluble egg proteins from PP = *Palomena prasina* with, from top to bottom, replicates 1 to 7. Spectra are shown baseline-subtracted, smoothed, with y-axis autoscaling, and covering the mass range 2 kDa to 20 kDa (with x-axis scale increments of 2 kDa).

Figure S8

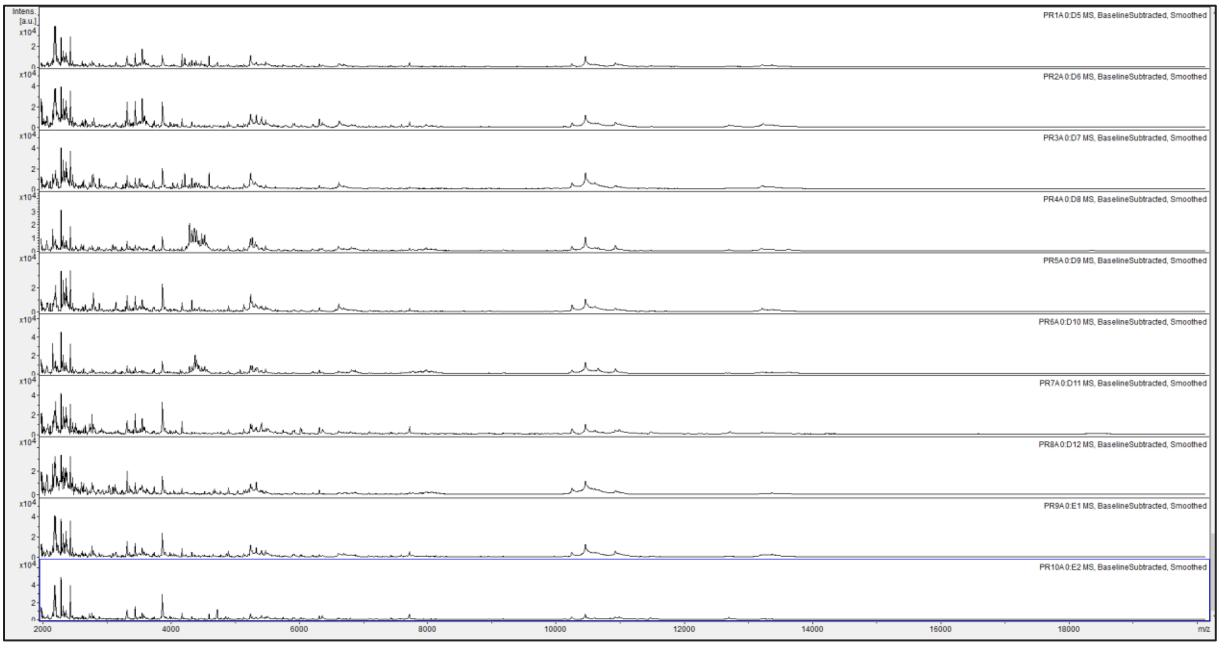

Figure S8 MALDI-TOF MS spectra of acid-soluble egg proteins from PR = *Pentatoma rufipes* with, from top to bottom, replicates 1 to 10. Spectra are shown baseline-subtracted, smoothed, with y-axis autoscaling, and covering the mass range 2 kDa to 20 kDa (with x-axis scale increments of 2 kDa).

Figure S9

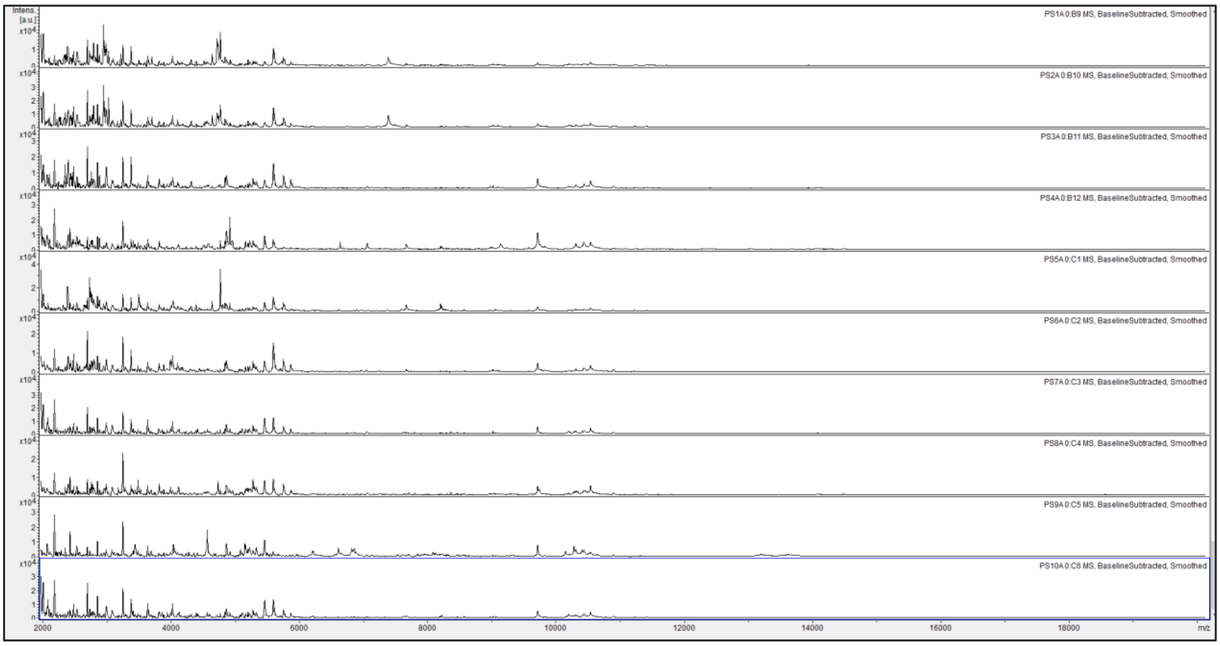

Figure S9 MALDI-TOF MS spectra of acid-soluble egg proteins from PS = *Peribalus strictus* with, from top to bottom, replicates 1 to 10. Spectra are shown baseline-subtracted, smoothed, with y-axis autoscaling, and covering the mass range 2 kDa to 20 kDa (with x-axis scale increments of 2 kDa).

Table S1 Bruker scores for comparisons between reference spectra (rows) and blind-test spectra (columns) subsequently identified as *Capocoris fuscispinus*, *Dolycoris baccarum*, and *Graphosoma italicum*.

	CF	CF	CF	CF	CF	CF	CF	CF	CF	CF	DB	DB	DB	DB	DB	DB	DB	DB	DB	DB	GI	GI	GI	GI	GI	GI	GI	GI	GI	GI
	X12	X19	X25	X32	X38	X48	X61	X74	X77	X86	X4	X5	X6	X9	X24	X31	X37	X71	X76	X83	X2	X7	X20	X26	X33	X39	X66	X79	X81	X84
CF10A	1.446	1.704	1.887	1.402	1.144	1.196	2.374	2.282	2.434	1.917	0.000	0.208	0.322	0.259	0.287	0.000	0.000	0.271	0.088	0.197	0.684	0.391	0.774	0.616	0.353	0.348	0.000	0.916	0.547	0.642
CF1A	1.363	2.228	1.560	0.850	0.864	1.147	1.478	1.716	1.650	1.623	0.079	0.665	0.543	0.610	0.610	0.639	0.681	0.526	0.000	0.756	0.615	0.050	0.690	0.476	0.511	0.664	0.518	0.649	0.579	0.025
CF2A	2.324	1.477	1.306	1.820	1.412	1.287	1.432	1.052	1.055	1.183	0.117	0.613	0.000	0.475	0.675	0.074	0.293	0.578	0.035	0.941	0.234	0.000	0.380	0.494	0.161	0.183	0.306	0.338	0.000	0.000
CF3A	1.291	1.676	2.152	1.315	1.017	0.999	1.516	1.472	1.758	2.262	0.784	0.360	0.142	1.043	0.475	0.249	0.498	1.046	0.000	0.609	0.625	0.344	0.336	0.009	0.000	0.073	0.000	0.048	0.425	0.375
CF4A	2.069	1.032	1.297	2.546	1.899	1.619	1.658	1.430	1.411	1.317	0.000	0.344	0.000	0.654	0.149	0.586	0.422	0.628	0.052	0.829	0.346	0.433	0.485	0.354	0.662	0.513	0.368	0.505	0.225	0.343
CF5A	1.518	1.376	1.738	1.618	1.230	1.579	2.039	2.077	1.733	1.571	0.000	0.812	0.369	0.992	0.184	0.282	0.495	0.219	0.836	0.543	0.335	0.927	0.000	0.000	0.182	0.451	0.545	0.423	0.627	0.103
CF6A	1.864	1.087	0.922	2.166	1.979	2.247	1.503	1.246	1.129	0.672	0.375	0.224	0.000	0.318	0.338	0.355	0.447	0.000	0.642	0.485	0.176	0.298	0.000	0.283	0.391	0.733	0.617	0.547	0.379	0.046
CF7A	1.881	1.121	0.769	2.195	2.602	1.348	1.261	1.243	1.168	0.633	0.112	0.000	0.031	0.603	0.572	0.388	0.452	0.449	0.140	0.493	0.033	0.749	0.364	0.370	0.417	0.188	0.000	0.317	0.000	0.240
CF8A	1.066	1.229	1.544	1.467	1.317	1.197	2.337	2.621	2.059	1.737	0.332	0.523	0.150	0.963	0.469	0.806	0.519	0.318	0.325	0.658	0.812	0.581	0.324	0.501	0.394	0.491	0.000	0.534	0.790	0.757
CF9A	1.080	1.575	2.386	1.175	1.041	0.370	1.464	1.525	1.610	2.545	0.689	0.348	0.795	0.993	0.679	0.930	0.856	0.524	0.000	0.420	0.757	0.000	0.906	0.582	0.092	0.589	0.442	0.000	1.005	0.541
DB10A	0.018	0.000	0.000	0.109	0.000	0.088	0.656	0.569	0.067	0.000	0.631	1.368	0.946	0.851	0.605	1.058	0.782	0.789	2.449	1.658	0.329	0.059	0.607	0.622	0.456	0.322	0.830	0.671	0.132	0.386
DB1A	0.601	0.895	0.181	0.180	0.134	0.000	0.299	0.419	0.000	0.163	1.222	1.369	1.227	1.509	1.063	2.466	1.728	1.656	0.832	1.368	0.000	0.000	0.586	0.467	0.000	0.446	0.557	0.000	0.000	0.000
DB2A	0.239	1.135	0.281	0.068	0.000	0.000	0.529	0.775	0.910	0.524	2.496	1.384	1.431	1.378	1.725	1.660	1.735	1.676	0.722	1.386	0.363	0.545	0.019	0.075	0.742	0.752	0.375	0.000	0.000	0.257
DB3A	0.518	0.737	0.528	0.736	0.412	0.150	0.127	0.282	0.065	0.197	1.125	2.510	1.847	1.590	1.554	1.244	1.097	1.554	1.401	1.941	0.377	0.534	0.767	0.660	0.864	0.579	0.750	0.743	0.000	0.000
DB4A	0.033	0.714	0.643	0.510	0.000	0.000	0.196	0.389	0.258	0.483	1.320	1.568	2.344	1.749	1.762	1.524	1.894	1.956	1.574	1.528	0.105	0.062	0.000	0.718	0.528	0.579	0.090	0.151	0.000	0.273
DB5A	0.000	0.418	0.885	0.775	0.835	0.902	0.120	0.005	0.000	0.457	1.096	1.976	1.766	1.420	1.616	1.407	1.284	1.649	1.417	2.549	0.220	0.107	0.000	0.000	0.624	0.216	0.552	0.392	0.000	0.263
DB6A	0.000	0.909	0.111	0.289	0.795	0.000	0.176	0.664	0.208	0.405	1.364	1.382	1.876	1.778	1.917	1.808	1.751	2.451	1.195	1.776	0.813	0.843	0.697	0.706	0.693	0.595	0.386	0.000	0.085	0.368
DB7A	0.000	0.641	0.120	0.723	0.502	0.000	0.272	0.753	0.883	0.819	0.971	1.528	1.933	2.592	1.455	1.640	1.501	1.899	1.281	1.684	0.000	0.122	0.886	0.000	0.394	0.311	0.327	0.300	0.000	0.000
DB8A	0.426	0.650	0.722	1.016	0.550	0.000	0.703	0.913	0.336	0.197	1.501	0.954	2.110	1.700	2.618	1.210	1.536	1.840	1.184	1.396	0.256	0.298	0.527	0.000	0.574	0.352	0.513	0.000	0.000	0.169
DB9A	0.451	0.489	0.119	0.558	0.505	0.000	0.554	0.530	0.000	0.645	1.281	0.945	1.882	1.635	1.569	2.027	2.618	2.089	1.213	1.356	0.353	0.000	0.114	0.490	0.376	0.136	0.213	0.397	0.256	0.252
GI10A	0.036	0.000	0.000	0.155	0.107	0.000	0.711	0.145	1.055	0.628	0.209	0.484	0.055	0.458	0.234	0.572	0.782	0.981	0.366	0.000	2.128	2.011	1.291	1.884	1.858	2.005	1.903	1.727	1.836	1.910
GI1A	0.452	0.548	0.000	0.374	0.461	0.606	0.678	0.486	0.582	0.815	0.327	0.667	0.535	0.692	0.171	1.036	0.714	1.020	0.875	0.500	1.728	2.016	1.292	2.181	1.900	2.609	2.112	1.993	1.823	1.700
GI2A	0.430	0.193	0.002	0.449	0.094	0.065	0.242	0.058	0.337	0.000	0.297	0.590	0.001	0.000	0.089	0.373	0.000	0.666	1.167	0.000	1.912	1.419	0.870	1.955	1.872	2.146	2.080	2.632	1.486	1.699
GI3A	0.550	0.071	0.000	0.000	0.000	0.000	0.228	0.420	0.254	0.518	0.000	0.595	0.147	0.590	0.314	0.432	0.297	0.589	0.771	0.222	1.634	1.136	1.906	1.846	1.902	1.469	2.008	1.682	1.596	1.592
GI4A	0.270	0.609	0.000	0.046	0.423	0.000	0.000	0.000	0.397	0.261	0.400	0.346	0.000	0.535	0.000	0.605	0.379	1.075	0.474	0.042	1.717	1.844	1.286	2.658	2.091	2.129	2.312	2.004	1.920	1.451
GI5A	0.602	0.234	0.000	0.002	0.000	0.000	0.594	0.151	0.000	0.000	0.517	0.552	0.669	0.536	0.130	0.193	0.234	0.800	0.000	0.429	1.920	1.725	1.107	2.032	2.625	1.788	2.121	1.809	2.093	1.879

GI6A	0.423	0.913	0.000	0.110	0.331	0.463	0.403	0.000	0.019	0.299	0.067	0.874	0.269	0.114	0.138	0.534	0.267	0.903	1.054	0.379	1.333	1.195	1.180	2.065	1.913	2.075	2.680	1.995	1.672	1.556
GI7A	0.507	0.000	0.114	0.577	0.080	0.051	0.000	0.000	0.000	0.505	0.020	0.398	0.518	0.223	0.254	0.000	0.662	1.018	0.000	0.306	2.621	1.600	1.574	1.537	2.024	1.611	1.693	1.825	2.046	2.016
GI8A	0.054	0.326	0.416	0.244	0.191	0.285	0.143	0.000	0.721	0.454	0.000	0.000	0.000	0.000	0.061	0.241	0.208	0.679	0.202	0.166	2.198	1.636	1.687	1.622	1.951	1.579	1.718	1.598	2.142	2.458
GI9A	0.593	0.638	0.535	0.655	0.306	0.000	0.439	0.184	0.426	0.626	0.000	0.389	0.214	0.635	0.000	0.092	0.430	0.953	0.000	0.000	2.135	1.753	1.461	1.912	2.013	1.440	1.739	1.547	2.506	2.116
HH10A	0.295	0.690	0.207	0.000	0.452	0.194	0.303	0.034	0.399	0.289	0.209	0.083	0.000	0.379	0.474	0.000	0.029	0.038	0.000	0.455	0.000	0.391	0.241	0.125	0.535	0.192	0.074	0.000	0.000	0.000
HH1A	0.303	0.826	0.279	0.000	0.506	0.000	0.432	0.373	0.561	0.259	0.270	0.209	0.134	0.835	0.024	0.000	0.169	0.219	0.567	0.000	0.000	0.344	0.640	0.000	0.451	0.000	0.281	0.667	0.414	0.271
HH2A	0.009	0.701	0.715	0.000	0.520	0.111	0.546	0.705	0.618	0.541	0.006	0.000	0.000	0.106	0.487	0.000	0.147	0.000	0.162	0.473	0.252	0.000	0.693	0.453	0.231	0.183	0.091	0.302	0.353	0.000
HH3A	0.608	0.438	0.427	0.228	0.744	0.119	0.437	0.378	0.802	0.604	0.409	0.000	0.000	0.222	0.493	0.000	0.000	0.000	0.215	0.520	0.000	0.510	0.223	0.315	0.443	0.304	0.050	0.634	0.271	0.000
HH4A	0.245	0.862	0.090	0.315	0.079	0.100	0.400	0.535	0.788	0.698	0.176	0.230	0.000	0.000	0.554	0.000	0.320	0.214	0.000	0.716	0.113	0.213	0.124	0.163	0.622	0.236	0.457	0.032	0.391	0.247
HH5A	0.542	0.897	0.198	0.000	0.415	0.000	0.688	0.371	0.512	0.458	0.038	0.000	0.000	0.326	0.000	0.000	0.000	0.000	0.193	0.000	0.222	0.000	0.384	0.463	0.241	0.220	0.799	0.000	0.294	0.012
HH6A	0.433	0.867	0.245	0.126	0.636	0.124	0.265	0.355	0.559	0.254	0.000	0.000	0.215	0.155	0.028	0.000	0.047	0.000	0.439	0.358	0.000	0.256	0.392	0.242	0.454	0.121	0.613	0.724	0.509	0.508
HH7A	0.306	0.592	0.158	0.030	0.424	0.000	0.087	0.038	0.674	0.260	0.000	0.000	0.000	0.266	0.255	0.335	0.357	0.000	0.430	0.346	0.000	0.709	0.111	0.376	0.429	0.216	0.276	0.257	0.653	0.000
HH8A	0.116	0.902	0.000	0.135	0.000	0.582	0.000	0.000	0.003	0.000	0.000	0.000	0.000	0.000	0.000	0.000	0.000	0.000	0.000	0.000	0.000	0.049	0.782	0.334	0.000	0.000	0.696	0.000	0.531	0.000
HH9A	0.298	0.965	0.110	0.000	0.223	0.000	0.000	0.207	0.691	0.461	0.547	0.296	0.514	0.000	0.198	0.000	0.587	0.627	0.009	0.627	0.008	0.232	0.841	0.000	0.671	0.657	0.219	0.000	0.042	0.000
NV10A	0.000	0.532	0.189	0.615	0.511	0.275	0.712	0.485	0.157	0.674	0.322	0.591	0.000	0.000	0.058	0.000	0.501	0.000	0.752	0.540	0.000	0.095	0.000	0.000	0.142	0.112	0.056	0.294	0.000	0.102
NV1A	0.796	0.000	0.452	0.343	0.413	0.431	0.000	0.000	0.000	0.698	1.016	0.752	0.579	0.207	0.599	0.628	0.000	0.971	0.292	0.360	0.000	0.803	0.000	0.010	0.613	0.000	0.153	0.220	0.705	0.000
NV2A	0.000	0.000	0.000	0.141	0.000	0.000	0.000	0.104	0.711	0.101	0.324	0.927	0.044	0.892	0.359	0.153	0.000	0.408	0.778	0.185	0.075	0.241	0.441	0.000	0.121	0.000	0.000	0.195	0.243	0.282
NV3A	0.000	0.439	0.213	0.263	0.288	0.463	0.596	0.280	0.453	0.436	0.374	0.773	0.615	0.464	0.228	0.346	0.720	0.385	0.805	0.728	0.179	0.189	0.000	0.013	0.310	0.293	0.039	0.250	0.225	0.659
NV4A	0.087	0.842	0.522	0.411	0.007	0.373	0.784	0.000	0.715	0.731	0.465	0.910	0.616	0.515	0.000	0.560	0.810	0.470	0.990	1.119	0.000	0.299	0.000	0.231	0.215	0.393	0.653	0.374	0.000	0.509
NV5A	0.199	0.764	0.448	0.569	0.000	0.853	0.947	0.494	0.805	0.610	0.000	0.964	0.000	0.093	0.404	0.525	0.000	0.617	0.985	0.956	0.000	0.048	0.078	0.000	0.418	0.000	0.286	0.360	0.169	0.369
NV6A	0.000	1.095	0.600	0.366	0.000	0.479	0.541	0.239	0.060	0.668	0.671	0.628	0.000	0.337	0.000	0.000	0.168	0.584	0.172	0.000	0.150	0.103	0.000	0.000	0.197	0.000	0.000	0.215	0.051	0.000
NV7A	0.081	0.277	0.000	0.000	0.000	0.072	0.000	0.000	0.355	0.288	0.000	1.167	0.424	0.130	0.034	0.225	0.085	0.342	0.857	0.506	0.000	0.273	0.084	0.395	0.421	0.000	0.000	0.384	0.000	0.404
NV8A	0.000	0.804	0.000	0.000	0.313	0.151	0.641	0.263	0.521	0.093	0.621	0.824	0.000	0.461	0.000	0.051	0.000	0.000	0.418	0.416	0.250	0.000	0.538	0.000	0.100	0.039	0.138	0.208	0.258	0.041
NV9A	0.000	0.621	0.295	0.000	0.324	0.001	0.374	0.000	0.133	0.253	0.432	0.794	0.000	0.260	0.545	0.000	0.006	0.561	1.118	0.284	0.205	0.000	0.000	0.000	0.000	0.000	0.121	0.668	0.000	0.218
PL10A	0.065	0.233	0.032	0.000	0.597	0.000	0.629	0.044	0.685	0.171	0.372	0.472	0.067	0.511	0.678	0.283	0.411	0.533	0.383	0.146	0.000	0.000	0.548	0.000	0.116	0.000	0.014	0.314	0.000	0.000
PL1A	0.000	0.619	0.000	0.000	0.000	0.527	0.145	0.537	0.646	0.054	0.000	0.000	0.000	0.000	0.000	0.000	0.220	0.000	0.000	0.000	0.000	0.783	0.000	0.000	0.211	0.000	0.820	0.821	0.000	0.042
PL2A	0.000	0.518	0.000	0.000	0.728	0.000	0.048	0.000	0.000	0.000	0.233	0.004	0.000	0.272	0.240	0.403	0.493	0.178	0.000	0.219	0.794	0.000	0.000	0.000	0.000	0.000	0.011	0.000	0.000	0.000
PL3A	0.059	0.232	0.461	0.061	0.877	0.031	0.453	0.195	0.445	0.278	0.274	0.145	0.000	0.734	0.816	0.429	0.385	0.298	0.478	0.026	0.367	0.085	0.111	0.000	0.102	0.000	0.000	0.033	0.000	0.000
PL4A	0.553	0.306	0.000	0.499	0.727	0.000	0.262	0.000	0.079	0.760	0.000	0.000	0.052	0.621	0.000	0.390	0.421	0.000	0.562	0.000	0.000	0.132	0.329	0.009	0.638	0.000	0.000	0.295	0.184	0.165
PL5A	0.000	0.145	0.000	0.000	0.923	0.000	0.000	0.496	0.246	0.171	0.030	0.244	0.154	0.000	0.000	0.280	0.488	0.000	0.000	0.000	0.000	0.492	0.148	0.000	0.085	0.000	0.000	0.000	0.120	0.000
PL6A	0.443	0.396	0.144	0.191	0.910	0.097	0.000	0.000	0.395	0.000	0.331	0.628	0.000	0.228	0.000	0.527	0.103	0.574	0.056	0.282	0.000	0.287	0.000	0.000	0.363	0.000	0.304	0.000	0.000	0.103
PL7A	0.047	0.107	0.263	0.000	0.689	0.000	0.359	0.196	0.293	0.205	0.130	0.342	0.000	0.260	0.272	0.433	0.484	0.381	0.167	0.647	0.000	0.000	0.000	0.000	0.000	0.000	0.000	0.000	0.000	0.038
PL8A	0.507	0.357	0.000	0.785	0.671	0.647	1.056	0.492	0.843	0.291	0.093	0.000	0.000	0.546	0.000	0.000	0.408	0.000	0.712	0.586	0.142	0.000	0.086	0.000	0.447	0.042	0.000	0.065	0.000	0.019
PL9A	0.158	0.291	0.000	0.271	0.382	0.000	0.636	0.338	0.206	0.450	0.348	0.107	0.000	0.513	0.318	0.209	0.419	0.376	0.295	0.310	0.066	0.000	0.000	0.291	0.000	0.000	0.000	0.000	0.000	0.000
PP1A	0.320	0.334	0.828	0.410	0.516	0.000	0.624	0.663	0.370	0.607	0.027	0.389	0.000	0.622	0.720	0.000	0.201	0.517	0.737	0.859	0.536	0.433	0.204	0.350	0.822	0.260	0.350	0.659	0.220	0.000
PP2A	0.432	0.535	0.374	0.237	0.639	0.085	0.000	0.079	0.000	0.131	0.518	0.000	0.000	0.429	0.000	0.000	0.327	0.561	0.367	0.405	0.000	0.123	0.000	0.396	0.000	0.729	0.000	0.000	0.000	0.141
PP3A	0.474	0.835	0.442	0.000	0.089	0.138	0.000	0.000	0.099	0.000	0.768	0.695	0.245	0.320	0.221	0.000	0.000	0.194	0.307	0.462	0.000	0.816	0.230	0.247	0.351	0.630	0.163	0.000	0.267	0.226
PP4A	0.413	0.606	0.100	0.097	0.181	0.176	0.000	0.000	0.703	0.000	0.900	0.053	0.000	0.000	0.513	0.043	0.301	0.000	0.734	0.089	0.000	0.331	0.000	0.206	0.000	0.000	0.177	0.510	0.000	0.000
PP5A	0.000	0.462	0.000	0.073	0.000	0.000	0.163	0.000	0.000	0.005	1.058	0.560	0.000	0.642	0.100	0.365	0.548	0.095	0.291	0.429	0.049	0.032	0.000	0.016	0.139	0.000	0.000	0.000	0.124	0.000
PP6A	0.585	0.206	0.000	0.290	0.071	0.399	0.000	0.000	0.000	0.000	0.541	0.010	0.000	0.076	0.392	0.134	0.076	0.318	0.724	0.000	0.000	0.531	0.000	0.260	0.000	0.278	0.000	0.182	0.000	0.000
PP7A	0.908	0.211	0.000	0.643	0.413	0.000	0.296	0.450	0.497	0.301	0.826	0.436	0.000	0.048	0.000	0.226	0.283	0.117	0.231	0.070	0.000	0.524	0.000	0.837	0.000	0.000	0.000	0.111	0.246	0.369
PR10A	0.006	0.183	0.504	0.000	0.007	0.786	0.000	0.101	0.412	0.000	0.184	0.134	0.000	0.000	0.515	0.210	0.335	0.000	0.000	0.314	0.000	0.000	0.339	0.229	0.080	0.000	0.467	0.000	0.459	0.605
PR1A	0.000	0.395	0.000	0.205	0.366	0.445	0.039	0.038	0.382	0.000	0.428	0.240	0.000	0.570	0.000	0.250	0.500	0.000	0.007	0.568	0.505	0.696	0.535	0.932	0.460	0.231	0.193	0.683	0.700	0.128
PR2A	0.046	0.110	0.000	0.100	0.000	0.886	0.295	0.541	0.472	0.462	0.716	0.285	0.851	0.440	0.000	0.084	0.411	0.304	0.385	0.627	0.000	0.440	0.000	0.429	0.207	0.000	0.000	0.293	0.406	0.000
PR3A	0.198	0.245	0.481	0.000	0.407	0.179	0.000	0.668	0.097	0.000	0.250	0.676	0.187	0.491	0.000	0.756	0.154	0.610	0.000	0.372	0.113	0.840	0.333	1.131	0.806	0.385	0.581	0.502	1.009	0.275
PR4A	0.000	0.835	0.000	0.484	0.277	0.000	0.261	0.556	0.444	0.000	0.033	0.765	0.357	0.615	0.075	0.417	0.187	0.389	0.774	0.608	0.489	0.232	0.000	0.245	0.000	0.000	0.576	0.448	0.272	0.283
PR5A	0.000	0.434	0.000	0.032	0.264	0.631	0.000	0.339	0.105	0.051	0.217	0.241	0.000	0.035	0.000	0.131	0.388	0.259	0.136	0.501	0.516	0.514	0.000	0.626	0.023	0.457	0.807	0.515	0.893	0.000
PR6A	0.248	0.524	0.404	0.289	0.037	0.010	0.513	0.641	1.074	0.000	0.192	0.572	0.158	0.735	0.207	0.169	0.472	0.000	0.348	0.728	0.000	0.372	0.123	0.221	0.329	0.191	0.000	0.169	0.231	0.000
PR7A	0.000	0.000	0.088	0.019	0.373	0.171	0.000	0.000	0.000	0.000	0.386	0.000	0.171	0.000	0.000	0.631	0.001	0.028	0.554	0.095	0.204	0.541	0.000	0.000	0.000	0.000	0.000	0.310	0.614	0.000
PR8A	0.040	0.693	0.093	0.000	0.201	0.274	0.070	0.156	0.011	0.000	0.766	0.351	0.010	0.706	0.457	0.466	0.646	0.601	0.000	0.358	0.000	0.471	0.000	0.284	0.560	0.003	0.000	0.000	0.786	0.466
PR9A	0.000	0.142	0.000	0.000	0.218	0.233	0.011	0.168	0.000	0.176	0.298	0.227	0.000	0.523	0.000	0.283	0.517	0.000	0.029	0.534	0.296	0.460	0.214	0.481	0.272	0.000	0.211	0.262	0.135	0.068
PS10A	0.877	0.596	0.705	1.016	1.122	0.606	0.310	0.131	0.646	0.446	0.558	1.115	0.228	0.353	0.659	0.000	0.000	0.130	0.696	0.688	0.000	0.000	0.000	0.000	0.000	0.336	0.534	0.372	0.166	0.681
PS1A	0.568	0.518	0.487	0.942	0.687	0.878	0.000	0.000	0.836	0.000	0.527	0.632	0.385	0.130	0.814	0.305	0.000	0.622	0.000	0.170	0.000	0.190	0.094	0.000	0.000	0.000	0.321	0.433	0.010	0.049
PS2A	0.441	0.666	0.554	0.799	0.653	0.546	0.000	0.000	0.433	0.076	0.409	0.674	0.283	0.000	0.173	0.072	0.000	0.575	0.000	0.000	0.379	0.490	0.000	0.000	0.000	0.211	0.273	0.023	0.000	0.458
PS3A	0.000	0.252	0.531	0.722	0.459	0.454	0.289	0.000	0.434	0.196	0.572	0.785	0.000	0.261	0.557	0.000	0.000	0.562	0.558	0.277	0.243	0.000	0.509	0.000	0.096	0.000	0.000	0.734	0.000	0.109
PS4A	0.442	0.000	0.000	0.460	1.222	0.454	0.000	0.416	0.484	0.000	0.543	0.818	0.184	0.000	0.000	0.000	0.000	0.459	0.073	0.657	0.510	0.002	0.000	0.523	0.508	0.692	0.774	0.416	0.640	0.320
PS5A	0.804	0.969	0.530	1.062	0.837	0.659	0.439	0.000	0.405	0.132	0.482	0.058	0.506	0.167	0.290	0.131	0.471	0.175	0.417	0.609	0.250	0.112	0.000	0.243	0.221	0.590	0.722	0.417	0.829	0.172
PS6A	0.734	0.678	0.426	0.642	1.165	0.032	0.751	0.000	0.887	0.333	0.732	0.918	0.405	0.294	0.184	0.031	0.583	0.621	0.651	0.347	0.000	0.581	0.540	0.000	0.000	0.000	0.149	0.866	0.609	0.547
PS7A	0.597	0.428	0.484	0.778	0.571	0.314	0.401	0.000	0.392	0.000	0.611	0.914	0.063	0.045	0.498	0.000	0.094	0.496	0.364	0.687	0.374	0.000	0.000	0.067	0.030	0.102	0.564	0.322	0.000	0.790
PS8A	0.000	0.426	0.507	0.853	0.858	0.523	0.097	0.000	0.596	0.451	0.409	0.742	0.060	0.486	0.652	0.000	0.549	0.687	0.610	0.773	0.153	0.554	0.488	0.000	0.240	0.000	0.000	0.490	0.242	0.171
PS9A	0.187	0.000	0.915	1.038	0.624	0.821	0.761	0.000	0.717	0.204	0.267	0.595	0.397	0.874	1.057	0.160	0.681	0.291	0.537	0.632	0.678	0.404	0.000	0.192	0.536	0.271	0.593	0.576	0.000	0.061

CF = *Capocoris fuscispinus*, DB = *Dolycoris baccarum*, GI = *Graphosoma italicum*, HH = *Halyomorpha halys*, NV = *Nezara viridula*, PL = *Piezedorus lituratus*, PP = *Palomena prasina*, PR = *Pentatoma rufipes*, and PS = *Peribalus strictus.*

Table S2 Bruker scores for comparisons between reference spectra (rows) and blind-test spectra (columns) subsequently identified as *Halyomorpha halys*, *Nezara viridula*, and *Piezedorus lituratus*.

	HH	HH	HH	HH	HH	HH	HH	HH	HH	HH	NV	NV	NV	NV	NV	NV	NV	NV	NV	NV	PL	PL	PL	PL	PL	PL	PL	PL	PL	PL
	X1	X3	X8	X29	X60	X65	X67	X78	X80	X82	X14	X15	X21	X27	X28	X34	X41	X59	X62	X70	X22	X30	X35	X42	X50	X54	X58	X63	X68	X73
CF10A	0.896	0.560	0.045	0.641	0.901	0.440	0.386	0.388	0.497	0.133	0.000	0.634	0.000	0.299	0.000	0.510	0.464	0.000	0.000	0.412	0.278	0.437	0.690	0.000	0.000	0.000	0.384	0.374	0.378	0.058
CF1A	0.404	0.664	0.378	0.942	0.391	0.930	0.868	0.840	0.000	0.067	0.223	1.003	0.000	0.935	0.679	0.656	0.514	0.577	0.000	0.535	0.000	0.468	0.279	0.032	0.000	0.326	0.813	0.000	0.000	0.510
CF2A	0.313	0.654	0.546	0.666	0.744	0.645	0.382	0.279	0.392	0.368	0.000	0.000	0.000	0.000	0.000	0.015	0.000	0.000	0.000	0.000	0.000	0.698	0.485	0.519	0.423	0.090	0.186	0.694	0.000	0.718
CF3A	0.898	0.415	0.233	0.355	0.662	0.712	0.638	0.198	0.000	0.246	0.087	0.252	0.000	0.308	0.000	0.554	0.000	0.123	0.000	0.039	0.000	0.143	0.226	0.208	0.000	0.000	0.694	0.319	0.301	0.414
CF4A	0.279	0.000	0.552	0.174	0.666	0.182	0.628	0.456	0.071	0.163	0.197	0.000	0.000	0.000	0.000	0.000	0.722	0.014	0.000	0.000	0.325	0.024	0.000	0.163	0.196	0.000	0.805	0.304	0.183	0.000
CF5A	0.598	0.000	0.566	0.000	0.832	0.357	0.048	0.000	0.428	0.617	0.230	0.623	0.000	0.427	0.374	0.329	0.848	0.000	0.302	1.166	0.608	0.535	0.665	0.474	0.531	0.156	0.659	0.568	0.792	0.717
CF6A	0.328	0.182	0.328	0.216	0.581	0.500	0.207	0.000	0.127	0.000	0.087	0.286	0.177	0.072	0.260	0.000	0.321	0.000	0.336	0.759	0.226	0.784	0.575	0.511	0.290	0.374	0.806	0.866	0.923	0.984
CF7A	0.690	0.037	0.267	0.456	0.509	0.371	0.698	0.000	0.112	0.052	0.000	0.000	0.000	0.000	0.330	0.152	0.018	0.000	0.000	0.000	0.510	0.000	0.719	0.273	0.554	0.627	1.147	0.922	0.625	0.475
CF8A	0.069	0.010	0.036	0.234	0.691	0.599	0.000	0.621	0.000	0.434	0.000	0.718	0.100	0.591	0.000	0.102	0.572	0.000	0.156	0.568	0.000	0.008	0.114	0.000	0.000	0.035	0.319	0.000	0.730	0.021
CF9A	0.629	0.153	0.046	0.136	0.383	0.338	0.256	0.456	0.000	0.000	0.000	0.422	0.000	0.000	0.027	0.654	0.249	0.449	0.000	0.391	0.000	0.000	0.272	0.000	0.000	0.000	0.539	0.437	0.000	0.000
DB10A	0.718	0.312	0.796	0.780	0.420	0.386	0.219	0.200	0.000	0.000	0.506	0.933	0.880	0.506	0.608	0.471	0.454	0.526	0.287	0.961	0.000	0.494	0.000	0.212	0.000	0.000	0.613	0.000	0.469	0.000
DB1A	0.323	0.137	0.290	0.000	0.000	0.264	0.103	0.123	0.000	0.058	0.271	0.751	0.464	0.824	0.076	0.606	0.782	0.000	0.000	0.941	0.012	0.662	0.762	0.467	0.133	0.107	0.644	0.485	0.773	0.464
DB2A	1.068	0.803	1.027	0.916	0.704	0.446	0.683	0.590	0.036	0.000	0.359	0.402	0.000	0.357	0.511	0.000	0.033	0.382	0.133	1.006	0.360	0.303	0.426	0.304	0.000	0.000	1.024	0.477	0.000	0.060
DB3A	0.305	0.000	0.419	0.453	0.000	0.000	0.102	0.479	0.000	0.000	0.717	0.890	0.805	0.406	0.715	0.234	0.824	0.681	0.854	0.564	0.000	0.000	0.309	0.000	0.060	0.175	0.614	0.319	0.179	0.000
DB4A	0.817	0.101	1.021	0.583	0.562	0.696	0.814	0.374	0.000	0.233	0.332	0.000	0.306	0.000	0.280	0.141	0.000	0.716	0.278	0.265	0.000	0.090	0.059	0.516	0.000	0.000	0.000	0.638	0.000	0.179
DB5A	1.114	0.671	0.753	0.938	0.516	0.000	0.954	0.992	0.125	0.000	0.342	0.366	0.151	0.395	0.541	0.624	0.506	0.825	0.437	0.992	0.000	0.243	0.734	0.492	0.000	0.532	0.499	0.584	0.621	0.000

DB6A	0.361	0.000	0.361	0.510	0.000	0.274	0.689	0.263	0.000	0.000	0.607	0.000	0.000	0.000	0.015	0.000	0.000	0.317	0.000	0.157	0.000	0.000	0.671	0.450	0.000	0.112	0.216	0.298	0.584	0.000
DB7A	0.377	0.409	0.557	0.356	1.075	0.560	0.433	0.188	0.000	0.414	0.000	0.527	0.905	0.532	0.000	0.000	0.863	0.000	0.000	0.683	0.000	0.372	0.591	0.532	0.000	0.618	0.733	0.593	0.257	0.719
DB8A	1.251	0.706	0.750	0.992	0.797	0.709	0.774	1.041	0.284	0.000	0.000	0.525	0.298	0.560	0.000	0.000	0.000	0.728	0.223	0.339	0.000	1.015	0.356	0.552	0.633	0.033	0.638	0.458	0.339	0.568
DB9A	0.312	0.000	0.419	0.193	0.698	0.329	0.191	0.178	0.000	0.000	0.377	0.231	0.000	0.000	0.081	0.231	0.073	0.118	0.000	0.559	0.000	0.332	0.375	0.226	0.000	0.118	0.292	0.209	0.000	0.000
GI10A	0.000	0.000	0.000	0.000	0.000	0.225	0.000	0.176	0.245	0.000	0.228	0.419	0.274	0.000	0.113	0.298	0.455	0.000	0.000	0.570	0.138	0.000	0.000	0.054	0.168	0.246	0.024	0.070	0.000	0.371
GI1A	0.118	0.230	0.956	0.000	0.335	0.130	0.506	0.000	0.000	0.000	0.000	0.795	0.000	0.000	0.621	0.508	0.000	0.000	0.091	0.259	0.000	0.000	0.449	0.000	0.000	0.000	0.114	0.000	0.000	0.000
GI2A	0.000	0.000	0.000	0.159	0.131	0.000	0.061	0.377	0.000	0.452	0.044	0.571	0.000	0.393	0.000	0.186	0.098	0.198	0.106	0.362	0.082	0.464	0.006	0.000	0.000	0.000	0.000	0.280	0.000	0.000
GI3A	0.728	0.303	0.457	0.000	0.001	0.286	0.419	0.030	0.000	0.305	0.342	0.423	0.240	0.000	0.036	0.730	0.000	0.000	0.000	0.050	0.000	0.075	0.000	0.000	0.453	0.461	0.143	0.000	0.000	0.057
GI4A	0.278	0.269	0.743	0.177	0.000	0.000	0.414	0.173	0.087	0.270	0.000	0.488	0.000	0.000	0.162	0.654	0.248	0.000	0.000	0.701	0.652	0.105	0.388	0.000	0.000	0.224	0.073	0.000	0.000	0.000
GI5A	0.000	0.310	0.495	0.241	0.070	0.000	0.022	0.207	0.000	0.000	0.000	0.390	0.000	0.000	0.326	0.000	0.000	0.000	0.000	0.000	0.000	0.252	0.303	0.000	0.000	0.142	0.081	0.000	0.000	0.005
GI6A	0.171	0.000	0.470	0.000	0.124	0.449	0.431	0.000	0.000	0.162	0.000	0.741	0.000	0.000	0.000	0.000	0.135	0.000	0.000	0.367	0.077	0.372	0.000	0.000	0.000	0.000	0.129	0.000	0.000	0.000
GI7A	0.112	0.501	0.000	0.230	0.000	0.204	0.000	0.000	0.000	0.273	0.276	0.000	0.191	0.000	0.373	0.120	0.000	0.502	0.000	0.000	0.032	0.000	0.000	0.000	0.000	0.000	0.106	0.198	0.000	0.106
GI8A	0.017	0.000	0.259	0.055	0.212	0.000	0.000	0.127	0.000	0.000	0.059	0.319	0.000	0.339	0.000	0.166	0.000	0.000	0.000	0.309	0.021	0.000	0.000	0.000	0.000	0.000	0.187	0.000	0.000	0.000
GI9A	0.403	0.056	0.436	0.000	0.185	0.536	0.153	0.000	0.000	0.521	0.445	0.000	0.000	0.000	0.000	0.266	0.000	0.028	0.000	0.000	0.377	0.034	0.000	0.448	0.000	0.451	0.421	0.000	0.000	0.000
HH10A	2.035	2.198	1.982	2.110	2.392	2.054	2.211	2.187	1.996	1.948	0.000	0.003	0.000	0.011	0.188	0.316	0.812	0.000	0.000	0.000	0.989	0.589	0.359	1.059	0.662	0.941	0.469	1.256	0.000	1.169
HH1A	2.125	2.351	2.585	2.286	2.049	2.066	2.290	2.338	2.175	1.989	0.000	0.000	0.000	0.000	0.000	0.244	0.072	0.251	0.000	0.022	0.970	0.680	0.499	1.156	0.869	1.091	0.300	1.246	0.000	0.771
HH2A	2.198	2.420	2.246	2.603	2.154	2.296	2.365	2.439	2.210	2.236	0.000	0.305	0.000	0.182	0.365	0.000	0.368	0.000	0.207	0.166	1.119	1.137	0.813	1.135	1.151	1.051	0.892	1.402	0.613	1.186
HH3A	2.219	2.319	2.236	2.268	2.334	2.253	2.619	2.279	2.263	1.957	0.000	0.051	0.000	0.016	0.000	0.000	0.035	0.000	0.227	0.034	1.027	1.018	0.535	1.211	1.063	1.209	1.027	1.365	0.314	1.189
HH4A	2.258	2.401	2.152	2.427	2.324	2.257	2.281	2.650	2.284	2.051	0.000	0.000	0.000	0.000	0.298	0.344	0.436	0.000	0.166	0.190	0.920	0.777	0.518	1.055	0.812	1.157	0.627	1.247	0.756	0.966
HH5A	2.155	2.433	2.230	2.435	2.203	2.574	2.289	2.376	2.074	2.071	0.000	0.000	0.000	0.000	0.000	0.000	0.473	0.000	0.000	0.059	1.002	0.968	0.657	0.917	1.020	1.248	0.886	1.369	0.031	1.122
HH6A	2.511	2.270	2.183	2.343	2.007	2.075	2.241	2.317	2.397	2.327	0.000	0.239	0.000	0.000	0.000	0.273	0.169	0.000	0.000	0.228	1.149	1.153	0.674	1.387	1.021	1.254	0.525	1.268	0.169	1.489
HH7A	2.112	2.150	2.096	2.281	1.906	2.155	2.095	2.178	2.285	2.783	0.000	0.872	0.160	0.000	0.000	0.000	0.204	0.000	0.000	0.000	1.358	1.417	1.018	1.439	1.251	1.409	0.871	1.371	1.121	1.360
HH8A	2.104	2.117	1.951	2.048	1.774	1.890	2.105	2.231	2.607	2.007	0.000	0.000	0.000	0.000	0.000	0.194	0.000	0.000	0.373	0.000	1.068	0.570	0.359	0.910	0.798	0.884	0.285	0.929	0.672	0.726
HH9A	2.163	2.677	2.143	2.401	2.110	2.200	2.215	2.413	2.129	2.119	0.000	0.000	0.000	0.196	0.000	0.184	0.378	0.000	0.000	0.000	1.106	0.951	0.333	1.110	0.919	1.351	0.960	1.285	0.180	1.259
NV10A	0.365	0.549	0.095	0.619	0.430	0.000	0.138	0.000	0.000	0.000	2.097	2.061	1.681	2.311	2.207	2.155	1.963	2.201	2.354	2.227	0.399	0.317	0.683	0.887	0.000	0.819	0.693	0.664	0.000	0.017
NV1A	0.594	0.542	0.486	0.424	0.276	0.959	0.465	0.413	0.324	0.000	2.107	1.953	2.323	2.359	2.156	1.852	1.735	2.194	2.352	1.853	0.000	0.457	0.871	0.495	0.028	0.000	0.506	0.275	0.374	0.202
NV2A	0.386	0.000	0.000	0.364	0.000	0.119	0.249	0.000	0.000	0.000	1.792	2.378	1.879	2.392	2.419	2.166	2.119	1.693	1.960	2.232	0.276	0.292	0.309	0.423	0.558	0.523	0.709	0.465	0.177	0.436
NV3A	0.000	0.003	0.000	0.418	0.836	0.865	0.600	0.201	0.000	0.000	2.007	2.222	1.696	2.150	2.490	2.279	2.620	1.677	1.844	2.403	0.047	0.511	0.682	0.375	0.273	0.675	0.737	0.406	0.012	0.136
NV4A	0.000	0.000	0.000	0.377	0.871	0.052	0.358	0.071	0.000	0.283	1.940	2.051	1.721	2.207	2.179	2.077	2.329	2.056	2.008	2.613	0.000	0.055	0.319	0.561	0.000	0.874	0.808	0.383	0.602	0.296
NV5A	0.265	0.000	0.000	0.321	0.317	0.219	0.066	0.000	0.041	0.000	2.116	2.219	2.040	2.267	2.423	2.194	2.085	1.945	2.075	2.227	0.000	0.353	0.613	0.268	0.000	0.439	0.662	0.480	0.565	0.000
NV6A	0.000	0.000	0.276	0.000	0.000	0.000	0.000	0.000	0.000	0.235	2.288	1.872	1.614	2.145	2.139	2.201	1.972	2.069	2.168	2.137	0.000	0.375	0.445	0.696	0.000	0.741	0.660	0.556	0.372	0.396
NV7A	0.321	0.010	0.000	0.080	0.753	0.393	0.693	0.039	0.000	0.000	2.114	1.957	2.206	2.242	2.439	2.236	2.147	1.905	2.277	2.081	0.000	0.577	0.411	0.494	0.120	0.280	0.399	0.248	0.122	0.188
NV8A	0.000	0.000	0.000	0.144	0.000	0.168	0.164	0.000	0.000	0.000	2.169	2.057	2.056	2.177	2.434	2.574	2.182	1.806	2.159	2.310	0.000	0.000	0.425	0.426	0.000	0.363	0.490	0.000	0.000	0.468
NV9A	0.588	0.000	0.000	0.000	0.000	0.018	0.164	0.083	0.089	0.000	1.895	1.828	1.461	2.061	2.056	2.117	1.917	1.921	2.244	1.971	0.000	0.175	0.841	0.779	0.000	0.866	0.507	0.805	0.360	0.000
PL10A	1.199	1.021	0.981	1.005	0.829	0.980	0.592	0.724	0.463	1.041	0.000	0.170	0.000	0.197	0.217	0.690	0.484	0.358	0.260	0.183	2.151	2.731	2.126	2.256	1.985	2.233	1.984	2.241	2.292	2.277
PL1A	1.704	1.096	1.368	1.340	1.113	1.150	1.206	1.145	1.309	1.111	0.497	0.000	0.975	0.000	0.520	0.329	0.258	0.725	0.000	0.155	2.143	2.013	1.869	1.881	1.751	1.713	2.184	1.643	2.009	2.133
PL2A	1.126	0.939	0.635	1.071	1.065	1.133	0.740	1.114	0.590	1.071	0.334	0.149	0.383	0.260	0.419	0.268	0.597	0.577	0.348	0.000	2.128	2.190	2.336	2.645	2.189	2.185	1.968	2.338	2.268	2.607
PL3A	1.261	0.991	0.602	1.145	0.836	1.095	0.723	0.785	0.469	1.014	0.289	0.684	0.497	0.357	0.505	0.291	0.683	0.588	0.296	0.097	2.245	2.213	2.281	2.412	2.260	2.239	2.003	2.601	2.077	2.494
PL4A	1.006	0.731	0.498	0.927	0.870	0.878	0.846	0.778	0.605	1.280	0.483	0.391	0.518	0.535	0.431	0.496	0.444	0.564	0.000	0.506	2.092	2.252	2.107	2.129	2.215	2.537	2.117	2.246	2.199	2.228
PL5A	1.101	0.862	0.407	1.189	0.927	1.245	1.142	1.019	0.884	1.043	0.053	0.000	0.458	0.000	0.237	0.195	0.695	0.000	0.000	0.007	2.037	2.022	2.238	2.421	2.598	2.281	1.750	2.300	2.042	2.207
PL6A	0.631	0.535	0.030	0.948	0.701	0.495	0.151	0.531	0.621	1.132	0.044	0.622	0.590	0.350	0.637	0.573	0.475	0.325	0.517	0.608	2.044	2.004	2.634	2.389	2.279	2.162	1.961	2.401	2.275	2.237
PL7A	1.129	0.980	0.872	1.086	0.893	1.099	0.874	0.471	0.434	1.167	0.058	0.303	0.539	0.165	0.286	0.095	0.644	0.087	0.237	0.162	2.303	2.202	2.327	2.592	2.269	2.232	2.103	2.415	2.226	2.753
PL8A	1.241	0.407	0.344	0.867	0.410	0.884	0.137	0.680	0.616	0.780	0.000	0.439	0.000	0.547	0.329	0.482	0.851	0.384	0.207	0.655	2.149	2.397	2.249	2.325	2.054	2.436	2.162	2.222	2.682	2.259
PL9A	1.431	1.184	1.246	1.306	0.998	1.320	1.260	1.066	0.730	1.200	0.339	0.478	0.344	0.390	0.645	0.551	0.731	0.710	0.498	0.367	2.359	2.450	2.322	2.527	2.322	2.536	2.059	2.492	2.387	2.433
PP1A	0.679	0.377	0.242	0.349	0.428	0.158	0.266	0.463	0.397	0.568	0.121	0.477	0.000	0.004	0.319	0.587	0.198	0.000	0.000	0.000	0.682	0.721	0.365	0.353	0.339	0.743	0.599	0.915	0.863	0.129
PP2A	0.367	0.000	0.313	0.126	0.302	0.204	0.463	0.000	0.392	0.471	0.108	0.000	0.226	0.000	0.268	0.276	0.136	0.000	0.000	0.123	0.504	0.208	0.549	0.349	0.000	0.591	0.237	0.862	0.806	0.000
PP3A	0.989	0.000	0.273	0.474	0.374	0.466	0.582	0.426	0.685	0.555	0.000	0.784	0.000	0.000	0.000	0.408	0.000	0.000	0.139	0.000	0.718	0.583	0.566	0.883	0.421	0.081	0.186	1.004	0.000	0.000
PP4A	0.827	0.628	0.533	0.416	0.197	0.279	0.000	0.404	0.567	0.336	0.054	0.000	0.305	0.527	0.303	0.000	0.000	0.000	0.000	0.875	0.571	0.923	0.801	0.522	0.520	0.615	0.684	1.034	0.769	0.235
PP5A	0.517	0.000	0.000	0.188	0.346	0.000	0.000	0.000	0.000	0.217	0.560	0.000	0.000	0.000	0.065	0.741	0.000	0.000	0.014	0.000	0.203	0.000	0.034	0.289	0.156	0.000	0.503	0.000	0.000	0.009
PP6A	0.800	0.779	0.090	0.728	0.377	0.470	0.401	0.579	1.049	0.935	0.000	0.000	0.653	0.000	0.000	0.000	0.000	0.000	0.273	0.860	0.515	0.779	0.149	0.602	0.674	0.836	0.117	0.696	0.318	0.000
PP7A	0.787	0.362	0.204	0.298	0.162	0.055	0.000	0.000	0.527	0.000	0.000	0.592	0.000	0.000	0.000	0.172	0.000	0.387	0.000	0.000	0.102	0.269	0.534	0.541	0.467	0.403	0.782	0.000	0.000	0.000
PR10A	0.044	0.000	0.012	0.000	0.490	0.058	0.343	0.000	0.000	0.000	0.175	0.261	0.000	0.422	0.803	0.552	0.700	0.113	0.301	0.819	0.000	0.000	0.000	0.010	0.000	0.000	0.000	0.164	0.000	0.000
PR1A	0.518	0.000	0.494	0.557	0.242	0.000	0.000	0.000	0.000	0.025	0.000	0.361	0.000	0.044	0.521	0.112	0.706	0.000	0.000	0.474	0.000	0.088	0.000	0.000	0.000	0.228	0.000	0.000	0.000	0.317
PR2A	0.760	0.000	0.898	0.000	0.311	0.327	0.056	0.000	0.067	0.000	0.000	0.339	0.000	0.000	0.404	0.117	0.678	0.000	0.000	0.521	0.013	0.232	0.000	0.000	0.000	0.000	0.514	0.188	0.232	0.000
PR3A	0.000	0.137	0.744	0.356	0.412	0.138	0.239	0.000	0.646	0.458	0.173	0.093	0.000	0.179	0.053	0.000	0.681	0.000	0.000	0.206	0.000	0.707	0.000	0.438	0.000	0.578	0.361	0.107	0.231	0.000
PR4A	0.000	0.337	0.371	0.328	0.181	0.366	0.175	0.000	0.098	0.000	0.000	0.648	0.000	0.333	0.427	0.527	0.970	0.000	0.000	0.434	0.000	0.351	0.151	0.217	0.000	0.661	0.763	0.583	0.736	0.212
PR5A	0.205	0.481	1.042	0.780	0.321	0.729	0.147	0.479	0.019	0.279	0.252	0.690	0.000	0.037	0.138	0.000	0.340	0.000	0.000	0.215	0.454	0.644	0.114	0.453	0.390	0.741	0.570	0.055	0.023	0.409
PR6A	0.000	0.000	0.069	0.000	0.416	0.192	0.000	0.215	0.000	0.000	0.000	0.416	0.000	0.096	0.945	0.762	0.880	0.000	0.000	0.636	0.265	0.268	0.000	0.055	0.029	0.719	0.827	0.620	0.529	0.000
PR7A	0.531	0.000	0.000	0.000	0.295	0.000	0.154	0.000	0.000	0.243	0.000	0.000	0.110	0.000	0.494	0.000	0.547	0.000	0.000	0.258	0.000	0.553	0.393	0.000	0.098	0.000	0.698	0.287	0.148	0.000
PR8A	0.307	0.277	0.966	0.231	0.415	0.000	0.188	0.267	0.697	0.359	0.051	0.165	0.000	0.033	0.284	0.276	0.278	0.000	0.022	0.000	0.000	0.231	0.150	0.000	0.000	0.534	0.642	0.092	0.696	0.100
PR9A	0.000	0.000	0.405	0.377	0.609	0.277	0.423	0.120	0.240	0.322	0.000	0.340	0.224	0.700	0.440	0.299	0.307	0.000	0.000	0.400	0.000	0.117	0.000	0.000	0.000	0.000	0.551	0.000	0.000	0.000
PS10A	0.496	0.225	0.771	0.103	0.679	0.213	0.263	0.805	0.461	0.480	0.490	0.380	0.302	0.450	0.000	0.574	0.455	0.309	0.000	0.602	0.002	0.657	0.160	0.506	0.310	0.258	0.574	0.541	0.253	0.133
PS1A	0.299	0.303	0.271	0.318	0.548	0.159	0.100	0.411	0.000	0.210	0.809	0.268	0.303	0.327	0.571	0.360	0.234	0.697	0.309	0.323	0.000	0.268	0.549	0.324	0.298	0.102	0.662	0.274	0.000	0.237
PS2A	0.345	0.204	0.399	0.447	0.818	0.399	0.360	0.066	0.064	0.312	0.392	0.000	0.065	0.349	0.312	0.260	0.125	0.490	0.351	0.279	0.000	0.514	0.389	0.772	0.151	0.308	0.569	0.375	0.170	0.231
PS3A	0.418	0.556	0.480	0.795	0.629	0.351	0.824	0.834	0.313	0.571	0.181	0.286	0.137	0.090	0.302	0.652	0.625	0.231	0.089	0.214	0.000	0.676	0.000	0.000	0.000	0.000	0.622	0.033	0.220	0.000
PS4A	0.421	0.243	0.399	0.184	0.377	0.000	0.400	0.834	0.000	0.278	0.548	0.076	0.302	0.000	0.026	0.000	0.000	0.380	0.202	0.182	0.000	0.238	0.000	0.082	0.000	0.000	0.000	0.356	0.000	0.039
PS5A	0.665	0.917	0.514	0.596	0.640	0.153	0.923	0.593	0.568	0.591	0.525	0.217	0.702	0.540	0.102	0.578	0.309	0.991	0.408	0.528	0.000	0.303	0.149	0.190	0.000	0.403	0.455	0.458	0.000	0.000
PS6A	0.609	0.544	0.666	0.625	0.318	0.000	0.504	0.876	0.534	0.600	0.206	0.000	0.565	0.332	0.476	0.263	0.386	0.000	0.046	0.000	0.000	0.215	0.428	0.420	0.458	0.000	0.445	0.246	0.470	0.206
PS7A	0.274	0.187	0.808	0.547	0.574	0.000	0.356	0.720	0.191	0.385	0.288	0.296	0.302	0.000	0.215	0.301	0.605	0.623	0.385	0.726	0.141	0.424	0.229	0.241	0.501	0.147	0.393	0.504	0.342	0.379
PS8A	0.059	0.021	0.218	0.000	0.341	0.000	0.268	0.000	0.000	0.184	0.443	0.557	0.000	0.792	0.614	0.604	0.832	0.691	0.669	0.648	0.000	0.684	0.284	0.286	0.153	0.718	0.593	0.383	0.000	0.310
PS9A	0.000	0.179	0.338	0.000	0.735	0.603	0.462	0.588	0.642	0.330	0.000	0.435	0.000	0.000	0.140	0.610	1.060	0.000	0.175	1.033	0.000	0.678	0.413	0.705	0.196	0.598	0.837	0.562	0.795	0.539

CF = *Capocoris fuscispinus*, DB = *Dolycoris baccarum*, GI = *Graphosoma italicum*, HH = *Halyomorpha halys*, NV = *Nezara viridula*, PL = *Piezedorus lituratus*, PP = *Palomena prasina*, PR = *Pentatoma rufipes*, and PS = *Peribalus strictus.*

Table S3 Bruker scores for comparisons between reference spectra (rows) and blind-test spectra (columns) subsequently identified as *Palomena prasina*, *Pentatoma rufipes*, and *Peribalus strictus.*

	PP	PP	PP	PP	PP	PP	PP	PR	PR	PR	PR	PR	PR	PR	PR	PR	PR	PS	PS	PS	PS	PS	PS	PS	PS	PS	PS	PP	PP	PP
	X10	X11	X18	X47	X52	X56	X69	X13	X40	X43	X49	X53	X57	X64	X72	X75	X87	X16	X17	X23	X36	X44	X45	X46	X51	X55	X85	X10	X11	X18
CF10A	0.000	0.553	0.826	0.000	0.299	0.535	0.324	0.736	0.094	0.212	0.000	0.000	0.782	0.000	0.508	0.003	0.234	0.721	0.179	0.000	0.303	0.277	0.087	0.289	0.410	0.355	0.832	0.000	0.553	0.826
CF1A	0.451	0.125	0.015	0.606	0.000	0.615	0.349	0.482	0.376	0.163	0.441	0.167	0.622	0.447	0.882	0.436	0.829	0.000	0.322	0.439	0.000	0.214	0.000	0.000	0.138	0.002	0.382	0.451	0.125	0.015
CF2A	0.430	0.736	0.434	0.274	0.000	0.665	0.000	0.540	0.000	0.850	0.552	0.000	0.436	0.000	0.387	0.104	0.721	0.875	1.019	0.139	0.425	0.565	0.415	0.616	0.676	0.614	1.145	0.430	0.736	0.434
CF3A	0.291	0.310	0.622	0.227	0.533	0.877	0.269	0.289	0.488	0.621	0.473	0.000	0.583	0.112	0.599	0.397	0.277	0.675	0.473	0.751	0.043	0.607	0.054	0.329	0.423	0.307	0.000	0.291	0.310	0.622
CF4A	0.189	0.608	1.006	0.741	0.000	0.544	0.432	0.475	0.000	0.337	0.321	0.068	0.000	0.000	0.000	0.485	0.328	0.941	0.865	0.733	0.983	1.345	0.715	0.826	0.629	0.697	0.671	0.189	0.608	1.006
CF5A	0.000	0.000	0.598	1.006	0.133	0.344	0.447	1.134	0.264	0.732	0.497	0.000	0.558	0.453	0.906	0.445	0.929	0.000	0.697	0.199	0.072	0.000	0.000	0.590	1.084	0.429	0.381	0.000	0.000	0.598
CF6A	0.000	0.000	0.000	0.548	0.434	0.176	0.000	0.000	0.000	0.221	0.506	0.000	0.000	0.000	0.000	0.000	0.160	0.794	0.752	0.933	0.892	0.581	0.366	0.000	0.896	0.442	0.615	0.000	0.000	0.000

CF7A	0.293	0.000	0.719	0.232	0.166	0.185	0.706	0.704	0.000	0.000	0.156	0.000	0.000	0.000	0.000	0.293	0.493	0.597	0.456	0.537	0.961	0.881	0.366	0.637	0.804	0.000	1.155	0.293	0.000	0.719
CF8A	0.000	0.156	0.489	0.000	0.279	0.000	0.435	0.673	0.766	0.580	0.000	0.808	1.122	0.738	0.092	0.553	0.748	0.474	0.272	0.000	0.387	0.000	0.008	0.000	0.366	0.000	0.532	0.000	0.156	0.489
CF9A	0.633	0.000	0.280	0.403	0.342	0.462	0.481	0.301	0.626	0.850	0.464	0.000	0.948	0.556	0.533	0.502	0.192	0.801	0.773	0.160	0.242	0.838	0.184	0.602	1.138	0.000	0.736	0.633	0.000	0.280
DB10A	0.617	0.208	0.384	0.860	0.629	0.428	0.201	0.228	0.477	0.397	0.077	0.592	0.408	0.335	0.957	0.716	0.791	0.363	0.779	0.465	0.672	0.344	0.836	0.208	0.775	0.644	0.697	0.617	0.208	0.384
DB1A	0.784	0.000	0.663	0.468	0.290	0.001	0.535	0.288	0.196	0.000	0.320	0.636	0.153	0.000	0.089	0.171	0.062	0.403	0.333	0.231	0.384	0.176	0.368	0.008	0.318	0.000	0.801	0.784	0.000	0.663
DB2A	0.444	0.920	0.577	0.535	0.505	0.569	0.490	0.461	0.000	0.264	0.222	0.228	0.382	0.185	0.418	0.265	0.707	0.347	1.140	0.974	0.599	0.460	0.391	0.000	0.932	0.451	0.267	0.444	0.920	0.577
DB3A	0.883	0.000	0.537	0.018	0.437	0.828	0.118	0.476	0.839	0.095	0.088	0.577	0.149	0.800	0.943	0.369	0.742	0.224	1.011	0.723	0.814	0.491	0.421	0.531	0.980	0.027	0.650	0.883	0.000	0.537
DB4A	0.031	0.253	0.000	0.323	0.000	0.000	0.000	0.574	0.725	0.112	0.341	0.862	0.283	1.071	0.377	0.459	0.014	0.712	0.530	0.060	0.549	0.867	0.527	0.000	0.000	0.000	0.531	0.031	0.253	0.000
DB5A	0.000	0.002	0.524	0.191	0.134	0.321	0.000	0.752	0.617	0.271	0.000	0.408	0.023	0.691	0.953	0.364	0.929	0.397	1.087	0.405	0.590	0.257	0.777	0.572	0.802	0.533	0.440	0.000	0.002	0.524
DB6A	0.194	0.239	0.174	0.508	0.209	0.302	0.000	0.658	0.520	0.000	0.563	0.640	0.774	0.237	0.182	0.087	0.637	0.400	0.588	0.000	0.677	0.270	0.509	0.572	0.436	0.027	0.048	0.194	0.239	0.174
DB7A	0.006	0.011	0.024	0.380	0.382	0.000	0.000	0.635	0.748	0.383	0.000	1.078	0.664	1.070	0.190	0.427	0.663	0.000	0.041	0.000	0.053	0.000	0.153	0.000	0.188	0.000	0.362	0.006	0.011	0.024
DB8A	0.000	0.000	0.081	0.186	0.167	0.365	0.000	0.029	0.334	0.277	0.136	0.548	0.627	0.618	0.511	0.143	0.429	0.612	0.718	0.403	0.309	0.224	0.245	0.758	0.000	0.000	0.764	0.000	0.000	0.081
DB9A	0.303	0.000	0.000	0.067	0.791	0.000	0.000	0.274	0.401	0.228	0.260	0.362	0.300	0.000	0.537	0.000	0.229	0.074	0.322	0.000	0.445	0.009	0.543	0.739	0.481	0.185	0.186	0.303	0.000	0.000
GI10A	0.013	0.425	0.000	0.000	0.517	0.000	0.000	0.080	0.110	0.000	0.427	0.672	0.287	0.950	0.000	0.661	0.801	0.262	0.000	0.018	0.000	0.000	0.000	0.122	0.000	0.000	0.407	0.013	0.425	0.000
GI1A	0.133	0.000	0.445	0.084	0.620	0.123	0.000	0.293	0.542	0.387	0.733	0.000	0.399	0.271	0.730	0.401	0.597	0.242	0.000	0.000	0.282	0.672	0.503	0.000	0.389	0.238	0.410	0.133	0.000	0.445
GI2A	0.603	0.447	0.345	0.000	0.727	0.000	0.000	0.000	0.000	0.123	0.050	0.000	0.217	0.302	0.006	0.203	0.536	0.455	0.354	0.615	0.421	0.231	0.990	0.000	0.765	0.498	0.164	0.603	0.447	0.345
GI3A	0.195	0.510	0.070	0.000	0.361	0.435	0.000	0.651	0.160	0.000	0.000	0.185	0.412	0.500	0.396	0.690	0.632	0.000	0.000	0.328	0.000	0.000	0.000	0.000	0.100	0.000	0.606	0.195	0.510	0.070
GI4A	0.870	0.506	0.239	0.406	0.737	0.116	0.326	0.704	0.396	0.908	0.486	0.000	0.379	0.303	0.112	0.527	0.386	0.228	0.000	0.000	0.388	0.230	0.717	0.000	0.290	0.000	0.976	0.870	0.506	0.239
GI5A	0.000	0.386	0.067	0.290	0.508	0.443	0.000	0.288	0.000	0.271	0.336	0.000	0.324	0.270	0.079	0.294	0.492	0.177	0.000	0.621	0.000	0.196	0.000	0.000	0.000	0.272	0.464	0.000	0.386	0.067
GI6A	0.243	0.077	0.434	0.000	0.508	0.000	0.000	0.491	0.000	0.172	0.310	0.089	0.000	0.576	0.000	0.255	0.298	0.420	0.000	0.114	0.009	0.366	0.553	0.000	0.216	0.000	0.471	0.243	0.077	0.434
GI7A	0.167	0.479	0.000	0.544	0.275	0.000	0.033	0.000	0.244	0.398	0.043	0.001	0.244	0.246	0.243	0.000	0.000	0.000	0.000	0.000	0.210	0.000	0.000	0.211	0.188	0.000	0.000	0.167	0.479	0.000
GI8A	0.000	0.403	0.000	0.000	0.470	0.163	0.000	0.022	0.365	0.268	0.366	0.000	0.270	0.000	0.599	0.045	0.218	0.030	0.022	0.084	0.535	0.000	0.479	0.000	0.000	0.000	0.000	0.000	0.403	0.000
GI9A	0.385	0.146	0.075	0.259	0.469	0.000	0.000	0.089	0.705	0.351	0.479	0.000	0.611	0.525	0.293	0.053	0.589	0.313	0.000	0.000	0.870	0.058	0.000	0.000	0.000	0.000	0.000	0.385	0.146	0.075
HH10A	0.662	0.401	0.687	0.693	0.410	0.690	0.391	0.628	0.597	0.000	0.127	0.602	0.000	0.465	0.427	0.404	0.632	0.651	0.942	0.755	0.769	0.822	0.584	0.000	0.913	0.372	1.068	0.662	0.401	0.687
HH1A	0.261	0.351	0.206	0.702	0.000	0.812	0.000	0.000	0.359	0.000	0.176	0.086	0.414	0.492	0.523	0.947	0.529	0.208	0.321	0.763	0.327	0.670	0.657	0.129	0.417	0.504	0.000	0.261	0.351	0.206
HH2A	0.898	0.550	0.579	0.275	0.696	0.885	0.166	0.549	0.398	0.000	0.397	0.000	0.000	0.556	0.000	0.150	0.674	0.306	0.355	0.962	0.000	0.760	0.514	0.000	0.713	0.000	0.990	0.898	0.550	0.579
HH3A	0.944	0.453	0.471	0.652	0.601	0.737	0.415	0.030	0.331	0.097	0.000	0.276	0.000	0.118	0.162	0.494	0.294	0.692	0.300	0.240	0.325	0.763	0.090	0.000	0.517	0.345	0.518	0.944	0.453	0.471
HH4A	1.039	0.646	0.432	0.395	0.546	0.943	0.479	0.426	0.107	0.000	0.028	0.000	0.000	0.000	0.338	0.076	0.556	0.425	0.427	0.472	0.000	0.710	0.193	0.000	0.161	0.000	0.750	1.039	0.646	0.432
HH5A	0.684	0.232	0.170	0.239	0.384	0.608	0.191	0.444	0.487	0.000	0.560	0.000	0.362	0.827	0.452	0.354	0.250	0.076	0.209	0.614	0.056	0.604	0.524	0.000	0.501	0.330	0.781	0.684	0.232	0.170
HH6A	0.945	0.304	0.284	0.388	0.831	0.731	0.153	0.000	0.000	0.000	0.115	0.428	0.339	0.179	0.551	0.100	0.415	0.296	0.843	0.688	0.218	0.546	0.198	0.000	0.422	0.694	1.056	0.945	0.304	0.284
HH7A	1.065	0.470	0.591	0.221	0.761	1.002	0.470	0.000	0.074	0.281	0.528	0.114	0.111	0.295	0.248	0.174	0.267	0.359	0.018	0.544	0.000	0.640	0.445	0.012	0.033	0.454	0.692	1.065	0.470	0.591
HH8A	0.613	0.158	0.000	0.295	0.397	0.763	0.644	0.000	0.443	0.000	0.280	0.313	0.297	0.112	0.076	0.181	0.000	0.211	0.459	0.000	0.000	0.335	0.000	0.003	0.103	0.718	0.989	0.613	0.158	0.000
HH9A	0.944	0.285	0.398	0.608	0.730	0.763	0.745	0.000	0.352	0.000	0.211	0.352	0.000	0.156	0.011	0.156	0.850	0.547	0.188	0.378	0.354	0.856	0.667	0.000	0.533	0.514	0.791	0.944	0.285	0.398
NV10A	0.000	0.303	0.000	0.089	0.000	0.113	0.281	0.362	0.403	0.864	0.106	0.509	0.722	0.691	0.801	0.419	0.969	0.176	0.194	0.489	0.677	0.511	0.016	0.117	0.587	0.519	0.694	0.000	0.303	0.000
NV1A	0.000	0.000	0.000	0.669	0.000	0.534	0.501	0.000	0.213	0.000	0.000	0.000	0.000	0.176	0.000	0.000	0.526	0.681	0.047	0.464	0.712	0.867	0.009	0.402	0.674	0.549	0.163	0.000	0.000	0.000
NV2A	0.000	0.092	0.518	0.123	0.000	0.362	0.386	0.000	0.665	0.235	0.000	0.177	0.396	0.327	0.451	0.094	0.522	0.389	0.000	0.496	0.307	0.000	0.000	0.615	0.538	0.401	0.156	0.000	0.092	0.518
NV3A	0.456	0.000	0.524	0.632	0.000	0.000	0.319	0.216	0.568	0.866	0.505	0.681	0.695	0.761	0.696	0.362	0.795	0.034	0.816	0.910	0.770	0.728	0.706	0.772	1.007	0.826	0.968	0.456	0.000	0.524
NV4A	0.000	0.506	0.045	0.259	0.291	0.016	0.000	0.399	0.084	0.975	0.203	0.880	0.458	0.778	0.725	0.789	0.944	0.338	0.082	0.725	0.000	0.337	0.365	0.204	0.516	0.581	0.768	0.000	0.506	0.045
NV5A	0.436	0.000	0.397	0.404	0.329	0.191	0.732	0.201	0.000	0.301	0.206	0.427	0.000	0.306	0.000	0.520	0.661	0.063	0.000	0.505	0.557	0.259	0.413	0.279	0.755	0.540	0.669	0.436	0.000	0.397
NV6A	0.333	0.266	0.267	0.000	0.000	0.085	0.000	0.647	0.322	0.509	0.408	0.063	0.689	0.009	0.005	0.000	0.567	0.547	0.055	0.538	0.711	0.739	0.784	0.000	0.398	0.000	0.151	0.333	0.266	0.267
NV7A	0.000	0.000	0.000	0.127	0.000	0.052	0.031	0.185	0.576	0.000	0.023	0.000	0.229	0.000	0.294	0.103	0.077	0.000	0.000	0.000	0.181	0.385	0.384	0.000	0.406	0.000	0.074	0.000	0.000	0.000
NV8A	0.000	0.315	0.000	0.000	0.000	0.000	0.000	0.440	0.633	0.854	0.399	0.439	0.608	0.453	0.656	0.546	0.773	0.303	0.008	0.000	0.577	0.141	0.250	0.319	0.102	0.144	0.332	0.000	0.315	0.000
NV9A	0.052	0.000	0.048	0.454	0.253	0.529	0.243	0.000	0.000	0.294	0.000	0.060	0.270	0.000	0.000	0.000	0.153	0.774	0.461	0.823	0.771	0.209	0.431	0.269	0.678	0.288	0.488	0.052	0.000	0.048
PL10A	0.000	0.386	0.076	0.551	0.511	0.645	0.396	0.252	0.775	0.780	0.660	0.433	0.788	0.390	0.778	0.989	0.377	0.678	0.480	0.731	0.548	0.146	0.202	0.833	0.581	0.481	0.638	0.000	0.386	0.076
PL1A	0.454	0.000	0.056	0.975	0.767	0.947	0.141	0.000	0.777	0.931	0.491	0.614	0.623	0.178	0.554	0.000	0.000	0.153	0.000	0.397	0.000	0.158	0.000	0.552	0.340	0.677	0.688	0.454	0.000	0.056
PL2A	0.000	0.000	0.076	0.285	0.674	0.252	0.000	0.000	0.494	0.498	0.485	0.534	0.467	0.662	0.609	0.809	0.213	0.615	0.465	0.289	0.339	0.016	0.000	0.000	0.284	0.107	0.586	0.000	0.000	0.076
PL3A	0.099	0.000	0.000	0.308	0.577	0.539	0.298	0.470	0.449	0.218	0.000	0.710	0.301	0.843	0.000	0.068	0.000	0.128	0.407	0.147	0.000	0.092	0.000	0.359	0.000	0.000	0.179	0.099	0.000	0.000
PL4A	0.221	0.000	0.000	0.440	0.369	0.352	0.613	0.539	0.817	0.698	0.127	0.534	0.656	0.712	0.616	0.867	0.527	1.072	0.680	0.203	0.268	0.399	0.000	0.296	0.711	0.000	0.912	0.221	0.000	0.000
PL5A	0.474	0.000	0.000	0.000	0.213	0.480	0.263	0.543	0.482	0.303	0.000	0.000	0.506	0.360	0.180	0.568	0.239	0.608	0.266	0.335	0.000	0.000	0.000	0.000	0.187	0.000	0.628	0.474	0.000	0.000
PL6A	0.639	0.002	0.000	0.299	0.408	0.637	0.567	0.000	0.215	0.273	0.097	0.000	0.381	0.000	0.327	0.523	0.000	0.752	0.306	0.000	0.180	0.000	0.000	0.000	0.344	0.148	0.573	0.639	0.002	0.000
PL7A	0.434	0.000	0.226	0.069	0.904	0.887	0.391	0.099	0.414	0.382	0.000	0.545	0.375	0.607	0.504	0.526	0.019	1.017	0.527	0.213	0.405	0.030	0.000	0.424	0.777	0.135	0.767	0.434	0.000	0.226
PL8A	0.524	0.101	0.005	0.576	0.730	0.277	0.533	0.496	0.610	0.403	0.000	0.668	0.546	0.596	0.522	0.532	0.529	0.306	0.498	0.224	0.000	0.000	0.000	0.346	0.003	0.000	0.549	0.524	0.101	0.005
PL9A	0.686	0.000	0.000	0.535	0.584	0.821	0.088	0.162	0.889	0.285	0.337	0.416	0.881	0.626	0.682	0.552	0.655	0.903	0.605	0.345	0.128	0.000	0.000	0.536	0.000	0.304	0.720	0.686	0.000	0.000
PP1A	1.670	1.121	1.814	2.120	1.889	1.516	1.436	0.243	0.071	0.269	0.648	0.000	0.415	0.000	0.538	0.304	0.159	0.509	0.094	0.000	0.306	0.677	0.663	0.256	0.279	0.423	0.498	1.670	1.121	1.814
PP2A	2.202	1.474	2.056	2.314	2.566	1.959	1.981	0.013	0.000	0.000	0.579	0.428	0.156	0.111	0.105	0.202	0.158	0.493	0.535	0.781	0.000	0.708	0.075	0.000	0.032	0.000	0.224	2.202	1.474	2.056
PP3A	2.071	1.645	1.790	2.101	2.093	2.385	1.990	0.000	0.534	0.360	0.742	0.000	0.655	0.440	0.888	0.448	0.511	0.987	0.682	0.516	0.626	0.956	0.712	0.000	0.561	0.209	0.908	2.071	1.645	1.790
PP4A	2.102	2.157	2.176	2.066	2.271	2.240	2.430	0.329	0.473	0.504	0.429	0.636	0.385	0.344	0.512	0.524	0.770	0.168	0.190	0.445	0.293	0.550	0.000	0.000	0.000	0.160	0.489	2.102	2.157	2.176
PP5A	2.270	1.932	2.486	2.022	2.069	2.042	2.081	0.131	0.630	0.420	0.325	0.441	0.277	0.418	0.000	0.471	0.000	0.940	0.094	0.375	0.718	0.248	0.531	0.000	0.000	0.592	0.002	2.270	1.932	2.486
PP6A	2.368	1.847	2.320	2.095	2.243	2.211	2.404	0.135	0.359	0.000	0.272	0.000	0.000	0.000	0.182	0.521	0.076	0.267	0.002	0.126	0.313	0.374	0.000	0.000	0.000	0.000	0.305	2.368	1.847	2.320
PP7A	1.764	2.152	1.847	1.525	1.775	1.865	2.148	0.000	0.820	0.248	0.676	0.300	0.211	0.000	0.308	0.563	0.818	0.482	0.392	0.110	0.314	0.724	0.709	0.000	0.531	0.000	0.304	1.764	2.152	1.847
PR10A	0.346	0.411	0.358	0.801	0.562	0.371	0.572	2.176	1.916	2.376	2.480	1.641	2.053	1.764	2.365	2.022	1.796	0.000	0.000	0.000	0.000	0.152	0.000	0.000	0.223	0.000	0.000	0.346	0.411	0.358
PR1A	0.630	0.000	0.446	0.619	0.121	0.429	0.393	1.976	2.098	2.136	2.249	1.554	2.162	1.889	2.596	2.188	1.854	0.122	0.329	0.570	0.366	0.298	0.244	0.000	0.824	0.471	0.283	0.630	0.000	0.446
PR2A	0.249	0.000	0.000	0.388	0.419	0.528	0.000	2.508	2.014	2.112	2.135	2.005	2.052	2.089	2.252	2.333	2.248	0.000	0.362	0.336	0.534	0.000	0.000	0.240	0.399	0.000	0.217	0.249	0.000	0.000
PR3A	0.429	0.301	0.410	0.154	0.521	0.718	0.125	1.884	2.451	1.997	2.035	1.699	2.371	1.920	2.324	2.007	1.751	0.041	0.588	0.413	0.385	0.632	0.000	0.036	0.312	0.435	0.000	0.429	0.301	0.410
PR4A	0.341	0.000	0.412	0.526	0.122	0.190	0.000	1.889	2.154	1.889	1.816	2.605	2.003	2.613	1.992	2.350	2.263	0.000	0.000	0.000	0.170	0.104	0.000	0.000	0.000	0.000	0.133	0.341	0.000	0.412
PR5A	0.394	0.000	0.232	0.410	0.410	0.163	0.207	2.111	2.325	2.324	2.086	1.984	2.548	1.951	2.278	2.185	1.892	0.021	0.422	0.377	0.000	0.000	0.000	0.259	0.506	0.569	0.456	0.394	0.000	0.232
PR6A	0.148	0.000	0.000	0.889	0.000	0.076	0.509	1.875	1.873	1.716	1.632	2.225	1.956	2.428	1.697	2.162	2.066	0.000	0.000	0.158	0.244	0.000	0.103	0.000	0.657	0.000	0.000	0.148	0.000	0.000
PR7A	0.206	0.578	0.362	0.000	0.077	0.548	0.103	2.267	2.018	2.591	2.441	1.751	2.130	1.945	2.338	2.264	1.863	0.000	0.000	0.165	0.000	0.249	0.099	0.000	0.169	0.000	0.000	0.206	0.578	0.362
PR8A	0.000	0.552	0.000	0.000	0.000	0.562	0.523	2.161	1.932	1.843	1.900	1.939	1.767	2.087	1.974	2.072	2.447	0.000	0.000	0.245	0.273	0.020	0.000	0.008	0.359	0.000	0.000	0.000	0.552	0.000
PR9A	0.553	0.000	0.000	0.000	0.203	0.472	0.000	2.254	2.157	2.236	2.178	2.006	2.285	2.153	2.392	2.454	2.187	0.000	0.000	0.000	0.000	0.000	0.000	0.000	0.350	0.000	0.007	0.553	0.000	0.000
PS10A	0.174	0.192	0.108	0.715	0.529	0.642	0.291	0.379	0.589	0.288	0.287	0.000	0.784	0.000	0.700	0.481	0.653	2.223	2.678	2.458	2.380	2.197	2.233	2.244	2.365	2.193	2.602	0.174	0.192	0.108
PS1A	0.731	0.880	0.893	0.697	0.669	0.778	0.186	0.000	0.000	0.380	0.190	0.000	0.633	0.000	0.617	0.000	0.000	2.605	2.129	2.110	2.189	2.623	2.052	2.000	2.136	1.801	1.999	0.731	0.880	0.893
PS2A	0.085	0.419	0.348	0.839	0.662	1.116	0.000	0.000	0.153	0.208	0.010	0.000	0.043	0.000	0.537	0.000	0.000	2.650	2.150	2.210	2.266	2.603	2.057	2.084	2.323	1.934	2.100	0.085	0.419	0.348
PS3A	0.444	0.571	0.000	0.545	0.303	0.537	0.756	0.304	0.000	0.103	0.055	0.113	0.514	0.051	0.780	0.025	0.159	2.107	2.405	2.669	2.431	2.125	2.288	2.174	2.446	2.326	2.232	0.444	0.571	0.000
PS4A	0.561	0.096	0.305	0.448	0.569	0.390	0.000	0.000	0.299	0.000	0.000	0.000	0.492	0.000	0.412	0.192	0.409	1.662	2.177	2.179	2.385	1.918	2.020	2.104	2.239	2.299	2.043	0.561	0.096	0.305
PS5A	0.417	0.000	0.480	0.250	0.119	0.444	0.000	0.392	0.020	0.485	0.394	0.000	0.454	0.000	0.473	0.251	0.654	1.940	2.065	1.992	2.147	2.150	2.557	1.991	2.018	1.924	1.921	0.417	0.000	0.480
PS6A	0.626	0.582	0.492	0.393	0.916	0.333	0.023	0.046	0.482	0.319	0.629	0.000	0.575	0.000	0.606	0.218	0.246	2.063	2.372	2.416	2.317	2.220	2.193	2.268	2.560	2.178	2.119	0.626	0.582	0.492
PS7A	0.305	0.292	0.000	0.636	0.327	0.575	0.000	0.000	0.417	0.071	0.000	0.148	0.289	0.000	0.343	0.301	0.643	2.073	2.763	2.368	2.229	2.120	2.119	2.158	2.290	2.188	2.591	0.305	0.292	0.000
PS8A	0.752	0.595	0.662	0.042	0.395	0.141	0.000	0.550	0.600	0.000	0.106	0.215	0.437	0.028	0.582	0.356	0.576	1.758	1.993	2.154	1.912	1.721	1.911	2.590	1.983	2.078	2.045	0.752	0.595	0.662
PS9A	0.142	0.254	0.000	0.649	0.415	0.029	0.000	0.314	0.458	0.464	0.438	0.596	0.746	0.676	0.123	0.752	0.988	1.281	1.810	1.878	1.518	1.661	1.613	1.575	1.599	1.502	1.748	0.142	0.254	0.000

CF = *Capocoris fuscispinus*, DB = *Dolycoris baccarum*, GI = *Graphosoma italicum*, HH = *Halyomorpha halys*, NV = *Nezara viridula*, PL = *Piezedorus lituratus*, PP = *Palomena prasina*, PR = *Pentatoma rufipes*, and PS = *Peribalus strictus*.

Table S4 Average Bruker scores for all 81 pairwise species comparisons.

		Test spectra								
		CF	DB	GI	HH	NV	PL	PP	PR	PS
Reference spectra	CF	1.552	0.419	0.385	0.371	0.218	0.335	0.340	0.364	0.471
	DB	0.378	1.569	0.326	0.409	0.372	0.290	0.273	0.413	0.412
	GI	0.274	0.380	1.847	0.165	0.160	0.088	0.230	0.294	0.188
	HH	0.350	0.167	0.271	2.233	0.093	0.929	0.531	0.252	0.419
	NV	0.318	0.418	0.169	0.182	2.109	0.366	0.179	0.359	0.397
	PL	0.277	0.240	0.100	0.909	0.359	2.239	0.334	0.432	0.309
	PP	0.250	0.302	0.187	0.364	0.151	0.427	2.022	0.321	0.335
	PR	0.206	0.302	0.302	0.233	0.238	0.205	0.287	2.104	0.149
	PS	0.474	0.388	0.258	0.399	0.351	0.287	0.397	0.283	2.145

CF = *Capocoris fuscispinus*, DB = *Dolycoris baccarum*, GI = *Graphosoma italicum*, HH = *Halyomorpha halys*, NV = *Nezara viridula*, PL = *Piezedorus lituratus*, PP = *Palomena prasina*, PR = *Pentatoma rufipes*, and PS = *Peribalus strictus*.

Table S5 Standard errors of Bruker scores for all 81 pairwise species comparisons.

		Test spectra								
		CF	DB	GI	HH	NV	PL	PP	PR	PS
Reference spectra	CF	0.476	0.290	0.261	0.273	0.280	0.303	0.274	0.311	0.344
	DB	0.313	0.454	0.272	0.347	0.313	0.275	0.271	0.286	0.300
	GI	0.258	0.317	0.353	0.201	0.216	0.150	0.233	0.245	0.248
	HH	0.270	0.211	0.239	0.183	0.164	0.351	0.265	0.231	0.306
	NV	0.287	0.337	0.193	0.245	0.220	0.271	0.209	0.299	0.286
	PL	0.280	0.224	0.195	0.307	0.232	0.215	0.286	0.268	0.287
	PP	0.257	0.281	0.234	0.266	0.241	0.311	0.294	0.247	0.289
	PR	0.243	0.250	0.277	0.252	0.271	0.254	0.237	0.240	0.199
	PS	0.328	0.289	0.259	0.253	0.256	0.236	0.282	0.259	0.283

CF = *Capocoris fuscispinus*, DB = *Dolycoris baccarum*, GI = *Graphosoma italicum*, HH = *Halyomorpha halys*, NV = *Nezara viridula*, PL = *Piezedorus lituratus*, PP = *Palomena prasina*, PR = *Pentatoma rufipes*, and PS = *Peribalus strictus*.

The data from Tables S4 and S5 are shown graphically in Figure 2.
